# The Asymmetric Influence of Emotion in the Sharing of COVID-19 Science on Social Media: Observational Study

**DOI:** 10.2196/37331

**Published:** 2022-12-08

**Authors:** Kai Luo, Yang Yang, Hock Hai Teo

**Affiliations:** 1 National University of Singapore Singapore Singapore; 2 University of Warwick Coventry United Kingdom

**Keywords:** COVID-19, science communication, emotion, COVID-19 science, online social networks, computational social science, social media

## Abstract

**Background:**

Unlike past pandemics, COVID-19 is different to the extent that there is an unprecedented surge in both peer-reviewed and preprint research publications, and important scientific conversations about it are rampant on online social networks, even among laypeople. Clearly, this new phenomenon of scientific discourse is not well understood in that we do not know the diffusion patterns of peer-reviewed publications vis-à-vis preprints and what makes them viral.

**Objective:**

This paper aimed to examine how the emotionality of messages about preprint and peer-reviewed publications shapes their diffusion through online social networks in order to inform health science communicators’ and policy makers’ decisions on how to promote reliable sharing of crucial pandemic science on social media.

**Methods:**

We collected a large sample of Twitter discussions of early (January to May 2020) COVID-19 medical research outputs, which were tracked by Altmetric, in both preprint servers and peer-reviewed journals, and conducted statistical analyses to examine emotional valence, specific emotions, and the role of scientists as content creators in influencing the retweet rate.

**Results:**

Our large-scale analyses (n=243,567) revealed that scientific publication tweets with positive emotions were transmitted faster than those with negative emotions, especially for messages about preprints. Our results also showed that scientists’ participation in social media as content creators could accentuate the positive emotion effects on the sharing of peer-reviewed publications.

**Conclusions:**

Clear communication of critical science is crucial in the nascent stage of a pandemic. By revealing the emotional dynamics in the social media sharing of COVID-19 scientific outputs, our study offers scientists and policy makers an avenue to shape the discussion and diffusion of emerging scientific publications through manipulation of the emotionality of tweets. Scientists could use emotional language to promote the diffusion of more reliable peer-reviewed articles, while avoiding using too much positive emotional language in social media messages about preprints if they think that it is too early to widely communicate the preprint (not peer reviewed) data to the public.

## Introduction

### Background

The COVID-19 pandemic has led to an unparalleled surge in global research publications on a single topic in documented history [1]. Research publications on COVID-19 accounted for roughly 8% of all PubMed research outputs in 2020 [1]. Such an incredible surge was seen in not only traditional scientific sources (eg, journals) but also preprint servers [1,2]. This uptake in research output coincides with the active social media engagement of COVID-19 science from the public [3]. The urgency and immediacy of pandemic information needs had promoted the use of social media for science communication among the public to an unprecedented level [1,4-7]. Understandably, the communication of COVID-19 science on social media is of critical importance because it could influence people’s behaviors and affect the effectiveness of government measures [5]. However, given the variance of scientific publications in terms of quality and the instantaneity of information transmission on social media, it is imperative for policy makers and scientists to understand what drives the diffusion of research publications on social media.

Communication of science to the public has traditionally relied on professionals (eg, journalists, scientists, and public health authorities) to meticulously translate scientific findings for public consumption [2]. Even in this professionally moderated communication context, prior studies have found that the virality of professionally articulated messages was strongly influenced by how they were framed [8]. For example, framing cancer research with an appropriate emotion can increase the public’s understanding, quality perception, trust, and engagement with the findings [9]. It is noteworthy that communication through social media, being both unmediated and spontaneous, provides a fertile ground that could augment the impact of emotion on content virality, especially during a crisis [10]. Indeed, recent studies in the COVID-19 context have shown that emotion-laded communication on social media could influence a wide-range of pandemic-related issues, such as vaccine communication, public health compliance, and preventive behavior [11-14]. Thus, we sought to investigate how the emotionality present in the text of social media messages about scientific publications on COVID-19 would influence their virality.

### Theoretical Background

Text-based emotions refer to the presence of fine-grained emotions, such as happy, sad, and angry, in human languages [15]. Prior research has found that text-based emotions in the form of emotion words or emotional framing of messages could affect people’s cognitive processing of the information in the context of written communication [16]. There have been 2 mainstream theoretical perspectives on emotions in prior studies [17]. One is the dimensional perspective that posits dimensions, such as valence and arousal, are the basic elements of emotions [18], and the other is the discrete perspective that considers discrete entities, such as happy, sad, anger, and fear, as the basic elements of emotions [19]. Prior literature has investigated the role of text-based emotions in online content sharing from different perspectives [20-23], and has provided competing theoretical explanations of how emotion influences content sharing. First, in social media engagement, people exhibit a social tendency to present a positive self-image for altruistic reasons (eg, to help others) or self-enhancement [24]. People are motivated to share things that make them look good or help signal their desired identities. Indeed, it is found that people are more likely to share positive scientific findings [8], positive New York Times articles [25], and positive marketing content [26,27]. Second, contrary to self-enhancement, there is also a “negativity bias” explanation [28,29]. It argued that, due to its evolutionary advantages, information involving negative emotions is generally found to be detected, processed, and transmitted faster than information involving positive emotions [20-23]. Content that aroused negative emotions was found to spread faster, especially in the domain of social media news, politics, and science conspiracy [30-33]. The third and perhaps most widely used theoretical explanation suggests that it is high-arousal emotions, whether of positive or negative valence, that contribute to online virality [34-37]. This perspective argues that beyond valence, emotions also differ in the level of psychological arousal or activation [38], and the psychological arousal and activation (or deactivation) of the emotion influence the transmissibility of the content [25].

Given the plurality of the emotional dynamics in social media sharing, we aimed to first establish which of the 3 theoretical explanations mentioned above is most likely true in the context of social media sharing of COVID-19 scientific research. Although self-enhancement motivation has been established in the context of the interpersonal sharing of professionally mediated science communication [8], the science behind the emerging phenomenon of sharing scientific findings about a novel infectious disease through large online social networks could be much more complex. On the one hand, the heightened situational uncertainty induced by the pandemic [39] could potentially lead to even stronger “negativity bias.” Recent studies found a heightened prevalence of negative emotions or a negative emotional climate on social media during the early months of the pandemic [10,40]. On the other hand, findings from early COVID-19 scientific research were arguably important information sources of pandemic news. Taking COVID-19 preprints as an example, although news media largely refrained from citing findings from preprints in their reports before the pandemic, the use of COVID-19 preprints became the new norm during the pandemic [2], and they were used in news articles at a rate almost 100 times that of non–COVID-19 preprints [41]. Would this “news-like” status combined with heightened situational uncertainty lead to more salient negativity bias in the diffusion of social media messages of COVID-19 science or would the emotional dynamics be dominated by high-arousal emotions, regardless of positive or negative emotions? More importantly, do the sources of the messages (eg, preprint servers vs peer-reviewed journals) lead to different emotional dynamics in their diffusion?

Peer-reviewed journal publications and preprints differ in their scientific uncertainty in that there is a possibility that the results may be invalidated by subsequent studies [42,43]. Although all studies carry some degree of scientific uncertainty, it is arguably much higher in preprints. A rigorous peer review and editorial process can help scrutinize and mitigate scientific uncertainty in most journal publications, but such a process is absent in preprints. This has led to heated debates over the virtue and danger of the use of preprints in science communication to the public [44-46]. However, partly due to the rare use of preprints in science communication to the public, it remains unknown whether social media messages about preprints exhibit a different pattern of diffusion from that of peer-reviewed journal publications. Moreover, to mitigate the influence of scientific uncertainty in the communication of any research, past studies have emphasized the moderator role of scientists [43]. Scientists are considered as important moderators in the communication of science to the public. Their expertise could facilitate better articulation on the significance and implication of scientific findings while clarifying the potential scientific uncertainty [43]. Yet, we have limited understanding of how the identity and emotions of scientists jointly influence the diffusion of social media messages of scientific research. Thus, we also investigated the extent to which scientist participation in the social media sharing of COVID-19 science influences the emotional dynamics.

### Research Questions

To address the above gaps in our knowledge, we collected all Twitter discussions of nearly 10,000 early (January to May 2020) COVID-19 English research articles in the life science and biomedical fields in both peer-reviewed journals and preprint servers from Altmetric. Altmetric provides quantification of the attention received online for an individual research article. It is increasingly being used as a research metric for science evaluation [47]. Using these data, we sought to address the following research questions:

1. What aspect of emotion (ie, positive valence, negative valence, or arousal) best explains the emotional dynamics in the social media sharing of COVID-19 scientific outputs?
2. Do the emotional dynamics of sharing have similar or divergent patterns between messages of preprint and peer-reviewed journal publications?
3. What are the emotional dynamics associated with the role of scientists as social media message creators in the sharing of COVID-19 science?

## Methods

### Data

To answer our research questions, we collected data from several sources. First, we obtained COVID-19–related medical English peer-reviewed journal publications, published prior to mid-May 2020, from the MEDLINE database (accessed through PubMed), where we retrieved each publication’s unique digital object identifier (DOI). We then used the PubMed application programming interface (API) to further retrieve each publication’s detailed metadata (ie, journal, title, category, authors, abstract, etc). Second, we extracted the DOIs of preprint medical publications in the same period from bioRxiv and medRxiv. We further used the bioRxiv API to extract all detailed metadata of each preprint. At the time of data collection, there were 6552 articles available on MEDLINE and 3725 articles from bioRxiv and medRxiv together. Third, social media mentions of all articles from the MEDLINE database and preprint servers were collected from Altmetric, a London-based commercial company that tracks, analyzes, and collects the online activity around scholarly outputs from a selection of online sources, such as blogs, Twitter, Facebook, Google+, mainstream news outlets, and media. We used a research fetch API to query the Altmetric database using DOIs. Fourth, because of Twitter’s terms of use, Altmetric could only share the status ID of tweets through their API. We further retrieved the details of each tweet through a Twitter developer account using the REST API.

The Altmetric collection of tweets contains original tweets, retweets, quoted tweets, and replies. We used original tweets and their retweets, which yielded a raw sample of 268,003 original tweets created before June 1, 2020. We further removed tweets from nonhuman accounts (eg, organizational accounts or bots) through (1) manually checking and matching all official Twitter accounts of each publisher, journal, and preprint server, and (2) manually checking accounts with excessively high tweet volume (>200 tweets) in our data. This resulted in a final sample of 243,567 original tweets and 729,319 retweets. See Multimedia Appendix 1 for more information on the raw data and the data cleaning process mentioned above [48,49]. Lastly, due to the fast-changing COVID-19 situation worldwide in the early months, we sought to collect situational data related to COVID-19 to serve as controls. More specifically, we further collected (1) daily worldwide COVID-19–confirmed cases and confirmed fatality data from a verified source, OurWorldInData, which is operated by the University of Oxford, and (2) daily global COVID-19 Twitter data [48].

By focusing on the early months (January to May 2020) of the COVID-19 pandemic, we generated a large corpus of original tweets (n=243,567) for analysis. Accordingly, our data covered 8612 articles from 1161 peer-reviewed journals in the MEDLINE database and 2 preprint servers (ie, bioRxiv and medRxiv) in the life science and biomedical fields (see Multimedia Appendix 1 for more details). Each tweet had a valid URL reference to the article, which was identified by a unique DOI, on either the journal or preprint website. Using the DOI, we could identify whether the article referred in the tweet was a *preprint* research article, a *peer-reviewed* research article, or an *opinion/letter* piece published in a peer-reviewed journal. Opinion/letter pieces include editorials, correspondence, letters, and comments. They are published individual opinions from esteemed members of the scientific community rather than research articles. They do not go through a peer-review process, but they also have a unique DOI. Correspondingly, we further constructed 3 subgroups of original tweets mentioning these different article types. The distribution of original tweets among these 3 different types of scientific articles was as follows: 47,570 tweets for preprint articles; 97,769 for peer-reviewed journal research articles; and 98,228 for journal opinion/letter pieces.

Our raw tweet data contained many non-English tweets as Altmetric collected those tweets based on the presence of valid URLs to the DOI-referenced articles instead of text keywords. To process these data, we wrote and used a simple detect-then-translate program, using a Google Translate API, to translate all non-English tweet texts, user screen names, and user biographies (self-described text descriptions) to English. The translated tweet texts were then used to generate variables in this research. Specifically, to quantify the emotion in each tweet, we first used the previously validated Linguistic Inquiry and Word Count (LIWC) dictionaries [49] of the affective process to count the presence of both positive (eg, important, positive, and hope) and negative (eg, fatal, lower, and critical) emotional words in the tweet text. The positive and negative dictionary word counts were generated using licensed LIWC 2015 software.

As mentioned earlier, the discrete perspective is also a critical theoretical approach to investigate emotions [19]. Thus, in addition to the valence of tweets, we wanted to take into account the discrete entities of emotions as well to provide a more comprehensive and robust view on the impact of emotions in the social media sharing of COVID-19 scientific outputs. To this end, we used a state-of-the-art machine learning algorithm trained in the tweet context (CrystalFeel) to gauge which of the 4 specific emotions (ie, joy, anger, fear, and sadness) was most salient in the tweet [50,51]. We sent the translated text corpus to the authors of CrystalFeel who returned the predicted label. Example tweets are provided in Table 1. Although multiple discrete emotions could appear in the same text concurrently, the algorithm is designed to output the most salient one based on an independently calculated intensity score for each individual emotion.

Lastly, content sharing was measured by the number of retweets. Because our data covered a relatively long timespan (ie, 5 months), we counted the number of retweets within a fixed period (eg, the first 168 hours [a week]) after the time of the tweet to make the retweet count of different tweets comparable.

Answering our third research question required us to identify scientists in related fields (ie, medical doctors or academic researchers in the life science and biomedical fields) among tweet message creators. Unfortunately, there was no reliable existing method for us to identify the relevant scientists. To ensure cost-effectiveness and maintain a focused research scope, we developed (and pilot tested) a 2-step classification approach that relied on keyword identification and heuristic rules. This rule-based algorithm extracted formal job titles (eg, clinician, doctor, physician, and surgeon) and related medical terms (eg, cardiology and gastroenterology) from the user screen name along with their text biography and then differentiated scientists from nonscientists. Our manual verification coding validated a 95.5% F1 score for the classification performance. We acknowledge that this method is imperfect as it can lead to underidentification of scientists. We estimated 30%-50% underidentification through manual validation of our classification results on random samples (Multimedia Appendix 2 [52,53]). Underidentification may result in an underestimation of the effect of scientists’ engagement. In other words, it may lead to more conservative estimation of the effect size; however, the direction of the estimated effect should be unbiased.

We further included a wide range of previously established control variables that capture the characteristics of the users, referenced articles, and COVID-19 pandemic situation. Table 2 provides descriptions of all variables used in this study, while Table 3 presents the summary statistics of all variables in the full sample as well as each subsample.

**Table 1:** Example tweets of each specific emotion.

Emotion	Tweet examples^a^
Joy	“Some more good news - In this cohort of patients hospitalized for severe Covid-19 who were treated with compassionate-use [DRUG], clinical improvement was observed in [NUMBER] of [NUMBER] patients. #coronavirus #COVID-19” “Good news. Large, retrospective [JOURNAL] study of n=[NUMBER]. [DRUG] did not increase risk of severe #COVID19.” “Some clinical important found about 2019-nCoV from [JOURNAL]. I picked up some important info and translate it Here.
Anger	“Are you serious? The stranger this gets the more it screams bioweapon. #COVID19 coronavirus male infertility” “The more vitamin D the less mortality from Coronavirus! The skin produces vitamin D with the sun. So why should we be locked up inside?” “I don't expect politicians to know understand the detail of science. But you can't insult science when you don't like it and then suddenly insist on something that science can't give on demand.”
Fear	“Horrific read about allocation of scarce medical resources with #COVID19 by [AUTHORS] in @[JOURNAL] - This is very sad and distressing.” “Severe COVID-19 complications: [SYMPTOM] may be observed in the acute phase in severe cases. Long-term [SYMPTOM] has been observed.” “Horrifying. Social distancing in [LOCATION] is almost next to impossible.”
Sadness	“Reading this here left me with depression without enough meme.” “Sadly, this new covid fact will be totally ignored and causing so many lives.” “First time I see a political editorial at the [JOURNAL]. And it is about the disaster that is happening in [COUNTRY]. So sad.”
Neutral	“Clinical Characteristics and Results of [TEST & SUBJECT] With COVID19.” “The present study provides ten key recommendations for the management of COVID-19 infections in [DISEASE GROUP]: #COVID19” “Here is the link of the last study on [DRUG]!”

^a^The URL has been removed.

**Table 2:** Descriptions of all variables.

Variable	Description
RT7D	# of retweets in the first 168 hours
preprint	=1 if the tweet source is a preprint article
peer	=1 if the tweet source is a peer-reviewed article
letter	=1 if the tweet source is a journal opinion/letter piece
scientist	=1 if the user is classified as a doctor or researcher in the life science and biomedical fields
liwc_positive	# of positive emotion dictionary words identified by LIWC^a^ 2015
liwc_negative	# of negative emotion dictionary words identified by LIWC 2015
emotion: joy	=1 if the tweet text is predicted to have a salient emotion of joy
emotion: anger	=1 if the tweet text is predicted to have a salient emotion of anger
emotion: fear	=1 if the tweet text is predicted to have a salient emotion of fear
emotion: sadness	=1 if the tweet text is predicted to have a salient emotion of sadness
emotion: neutral	=1 if the tweet text is predicted to have no specific emotion
log_follower	(log) number of followers the user had
verified	=1 if the user is a verified user
length	# of words in the tweet text
hashtags	# of hashtags used in the tweet
mention	=1 if the tweet contains any mention of other users
title_length	# of words in the reference article in preprints or journal
title_liwc_pos	# of positive emotion words in the title identified by LIWC 2015
title_liwc_neg	# of negative emotion words in the title identified by LIWC 2015
log_cov_tweet	(log) rolling 7-day total number of global coronavirus tweets
log_cov_case	(log) rolling 7-day total number of global new confirmed COVID cases
log_cov_fatality	(log) rolling 7-day total number of global new confirmed COVID fatalities

^a^LIWC: Linguistic Inquiry and Word Count.

**Table 3:** Summary statistics of all variables.

Variable	Combined sample (N=243,567), mean (SD)	Preprint (N=47,570), mean (SD)	Peer-reviewed article (N=97,769), mean (SD)	Journal letter (N=98,228), mean (SD)
RT7D^a^	4.928 (85.873)	6.351 (75.654)	5.022 (87.606)	4.145 (88.729)
preprint	0.195 (0.396)	N/A^b^	N/A	N/A
peer	0.401 (0.490)	N/A	N/A	N/A
letter	0.403 (0.491)	N/A	N/A	N/A
scientist	0.183 (0.387)	0.156 (0.363)	0.179 (0.383)	0.201 (0.401)
liwc_positive	0.324 (0.634)	0.316 (0.619)	0.300 (0.614)	0.352 (0.661)
liwc_negative	0.206 (0.506)	0.208 (0.498)	0.191 (0.480)	0.221 (0.535)
emotion: joy	0.245 (0.430)	0.280 (0.449)	0.248 (0.432)	0.225 (0.417)
emotion: anger	0.050 (0.219)	0.045 (0.207)	0.034 (0.181)	0.070 (0.255)
emotion: fear	0.410 (0.492)	0.400 (0.490)	0.416 (0.493)	0.409 (0.492)
emotion: sadness	0.026 (0.159)	0.021 (0.143)	0.021 (0.145)	0.033 (0.179)
emotion: neutral	0.269 (0.443)	0.254 (0.435)	0.281 (0.449)	0.264 (0.441)
log_follower	6.367 (2.174)	6.345 (2.266)	6.329 (2.205)	6.415 (2.096)
verified	0.039 (0.194)	0.038 (0.191)	0.039 (0.194)	0.039 (0.194)
length	19.661 (12.893)	21.477 (13.021)	19.969 (12.807)	18.475 (12.796)
hashtags	0.648 (1.387)	0.647 (1.378)	0.667 (1.428)	0.630 (1.350)
mention	0.200 (0.400)	0.176 (0.381)	0.201 (0.401)	0.211 (0.408)
title_length	11.060 (4.733)	13.051 (5.063)	12.511 (4.303)	8.652 (3.859)
title_liwc_pos	0.101 (0.322)	0.074 (0.280)	0.090 (0.301)	0.125 (0.359)
title_liwc_neg	0.087 (0.289)	0.087 (0.290)	0.103 (0.311)	0.070 (0.262)
log_cov_tweet	15.829 (0.166)	15.817 (0.138)	15.831 (0.176)	15.834 (0.168)
log_cov_case	12.507 (1.283)	12.530 (1.300)	12.504 (1.346)	12.498 (1.209)
log_cov_fatality	9.690 (1.530)	9.740 (1.562)	9.681 (1.611)	9.674 (1.428)

^a^RT7D: number of retweets in the first 168 hours.

^b^N/A: not applicable.

### Statistical Analysis

To answer each of our research questions, we examined (1) the impacts of positive versus negative emotional language; (2) the impacts of specific emotions, such as joy, anger, fear, and sadness; and (3) the role of scientists as social media message creators in sharing about COVID-19 medical scientific papers through statistical analysis. We referred to the collective findings from answering these questions as the emotional dynamics in sharing COVID-19 science on social media. Because the distribution of the retweet count was highly skewed (see Table 3), we fitted a negative binomial regression with a maximum likelihood estimator, which is the most appropriate for data with overdispersion. This method is consistent with prior studies using Twitter data [37]. To further ensure that we obtained an unbiased standard error for statistical inference, we used clustered robust standard error [54] at the article level to account for and correct potential intracluster error correlation.

Consistent with prior studies [8], we estimated models both with and without article-level fixed effects. Models without fixed effects capture the between-article comparison, while models with fixed effects provide within-article comparison. The article-level fixed effect, or within-article effect, results were obtained using unconditional fixed effect negative binomial estimators [55]. More specifically, article dummies were included in the regression model to obtain the unconditional fixed effect results. Lastly, we assessed the robustness of our results under 2 criteria: (1) an alternative window for counting retweets (eg, 48 hours after the original tweet rather than a week), and (2) an alternative statistical model, that is, a zero-inflated negative binomial model, to account for the excessive presence of zeros in the retweet count. We showed that our key findings were highly robust under these criteria. More details are discussed and reported in Multimedia Appendix 3 [56,57].

Lastly, to buttress any findings from the statistical analysis on the effect of positive and negative emotion words in tweet text, we further conducted explorative analyses using a word cloud plot. We created 4 text corpuses along the emotion dimension (ie, positive vs negative) and tweet source dimension (ie, preprint vs peer reviewed). For example, if a *positive* dictionary word identified using LIWC 2015 appeared in tweet or retweet text (the text in the retweet was exactly the text in the original tweet being retweeted) about a preprint, this word was added to the *positive* *preprint* text corpus. Then, each word in the 4 text corpuses was processed to keep only the word stem and the term frequency-inversed document frequency weight for each word in the text corpuses to create the word cloud. More details on the text processing and word cloud creation process are provided in Multimedia Appendix 1.

### Ethical Considerations

This paper uses only secondary public data from an authorized Twitter commercial data vendor in compliance with Twitter privacy policy. Apart from the public Twitter handle, our data do not contain any individual identifier.

## Results

### Positive Versus Negative Language

We started with positive and negative emotional language. In the combined sample of all original tweets, our regression analysis (see Multimedia Appendix 4) revealed a significant main effect of positive emotional language on retweet rate (incidence rate ratio [IRR] 1.075, 95% CI 1.027-1.125; *P=*.002) but not for negative emotional language (IRR 1.015, 95% CI 0.953-1.082; *P=*.64). The results implied that one additional positive emotional word in a tweet mentioning a COVID-19 research article was associated with, on average, a 7.5% higher retweet rate, while a negative emotional word had a neutral impact. It highlighted that positivity spreads faster than negativity in the Twitter sharing of COVID-19 research, implying the existence of a “positivity bias” rather than a “negativity bias,” where positive emotion was found to spread faster. Further, the moderation test between LIWC emotional dictionary word counts and tweet source indicators revealed a positive interaction effect between the positive emotional word count and preprint indicator (IRR 1.129, 95% CI 1.034-1.233; *P=*.007), implying that an additional positive emotional word would increase the retweet rate difference between tweets mentioning preprint research and peer-reviewed research by 12%, while all other interactions remained insignificant. This points to a differential effect of the presence of emotion in tweets about different scientific sources. Thus, we next examined the effects of positive and negative emotional language separately on each subgroup to check if this pattern persisted in all 3 subgroups of tweets mentioning different types of articles (see Models 1-3 in Table 4).

The above results suggested that the “positivity bias” was only prevalent and visible in tweets that mentioned COVID-19 preprints. To further check the findings’ robustness, we also analyzed the within-article effects following a past study on the interpersonal sharing of science to the public [8]. Specifically, we used fixed effects to control for the articles’ influence on retweet count. As shown in Models 4-6 in Table 4, the within-article effects were largely consistent with the previously observed pattern. Only the positive word count in the preprint subgroup was found to significantly increase the retweet count. All other estimated coefficients of positive and negative emotional words remained insignificant.

**Figure 1:**
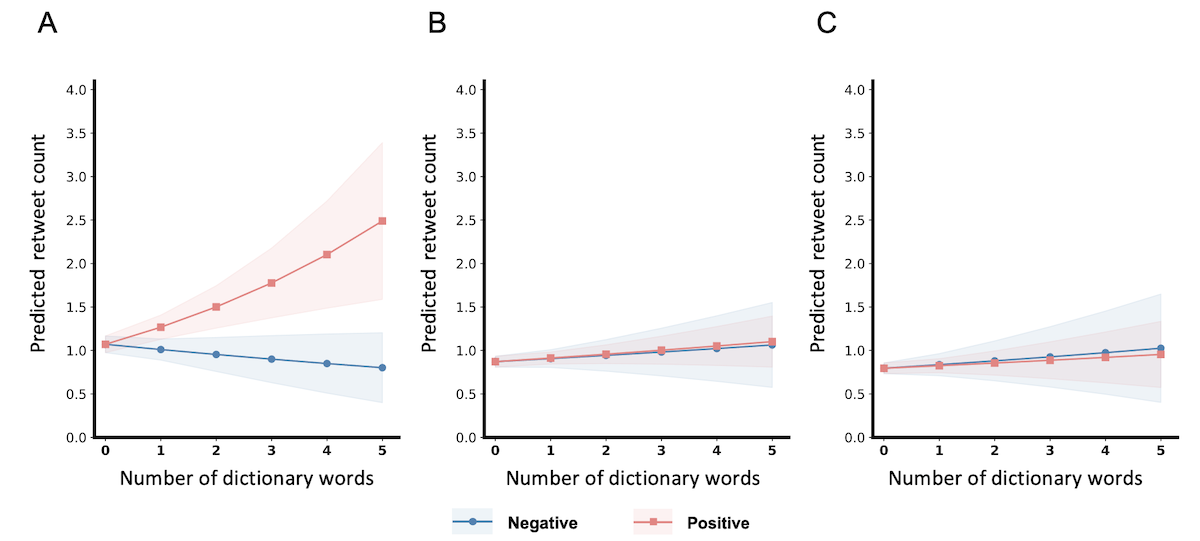
Prediction of the retweet count for (A) preprints, (B) peer-reviewed articles, and (C) journal letters. Positive emotion Linguistic Inquiry and Word Count dictionary words in tweets about preprints predict the highest retweet count. Bands indicate the 95% CIs.

Our results implied that there were divergent patterns among these 3 subgroups. More specifically, the “positivity bias” was only present in tweets mentioning preprints, which predicted that one additional positive emotional word was associated with a 17.7% increase in the retweet rate (IRR 1.177, 95% CI 1.089-1.272; *P<*.001), while the effect of a negative word was neutral (IRR 0.980, 95% CI 0.883-1.088; *P=*.70; see Figure 1 for a graphical illustration). In tweets mentioning either research articles or opinion/letter pieces in peer-reviewed journals, neither positive emotional words (research article: IRR 1.048, 95% CI 0.990-1.110; *P=*.11; opinion/letter pieces: IRR 1.043, 95% CI 0.952-1.143; *P=*.37) nor negative emotional words (research article: IRR 1.033, 95% CI 0.944-1.131; *P=*.47; opinion/letter pieces: IRR 1.041, 95% CI 0.936-1.158; *P=*.45) had statistically significant effects on the retweet rate.

**Figure 2:**
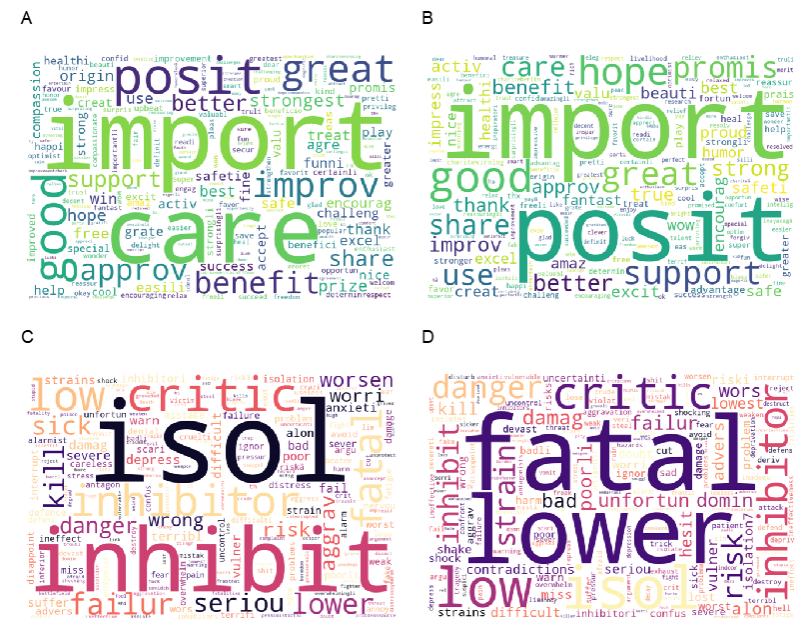
Word cloud plot of all positive/negative emotional words in tweets about preprints and peer-reviewed articles (word size indicates the term frequency-inversed document frequency weight). (A) positive–peer-reviewed articles; (B) positive–preprints; (C) negative–peer-reviewed articles; (D) negative–preprints.

Although the results of the statistical analyses implied the existence of a “positivity bias,” they cannot explain why it exists. Hence, we sought to further provide some explorative insights. Using word cloud plots (Figure 2), we showed that the positive words in tweets about preprints had a higher concentration of words like “hope,” “support,” and “promise” than tweets about peer-reviewed research (see Multimedia Appendix 5 for the exact weight difference). According to the psychological meaning of words [49], besides the positive affective process, the other categories shared by at least two of these three words were “verb,” “cognitive process,” and “present focus.” Qualitatively, these aspects could further elicit a sense of action alongside positivity, which could be a key positivity aspect that people seek under adverse circumstances, such as the COVID-19 crisis.

**Table 4:** Negative binomial estimation results using the Linguistic Inquiry and Word Count emotional dictionary word counts in subgroups.

Variable			Model 1 (preprint; N=47,570)^a,b^		Model 2 (peer reviewed; N=97,769)^a,b^		Model 3 (journal letter; N=98,228)^a,b^		Model 4 (preprint; N=47,570)^a,c^		Model 5 (peer reviewed; N=97,769)^a,c^		Model 6 (journal letter; N=98,228)^a,c^
**liwc_positive**													
	IRR^d^	1.177^e^		1.048		1.043		1.084^f^		1.048^g^		1.029	
	SE^h^	0.047		0.030		0.049		0.036		0.027		0.025	
**liwc_negative**													
	IRR	0.980		1.033		1.041		1.031		1.030		1.032	
	SE	0.052		0.047		0.056		0.040		0.043		0.032	
**log_follower**													
	IRR	1.785^e^		1.879^e^		1.891^e^		1.930^e^		1.915^e^		1.933^e^	
	SE	0.059		0.027		0.026		0.025		0.022		0.024	
**verified**													
	IRR	2.040^e^		1.865^e^		1.465^e^		2.003^e^		2.032^e^		1.822^e^	
	SE	0.283		0.223		0.202		0.196		0.210		0.275	
**length**													
	IRR	1.049^e^		1.050^e^		1.051^e^		1.055^e^		1.053^e^		1.049^e^	
	SE	0.004		0.002		0.004		0.002		0.002		0.002	
**hashtags**													
	IRR	1.042^f^		1.037^e^		1.012		1.064^e^		1.032^e^		1.018	
	SE	0.017		0.013		0.016		0.015		0.011		0.018	
**mention**													
	IRR	1.944^e^		1.604^e^		1.703^e^		1.601^e^		1.469^e^		1.632^e^	
	SE	0.149		0.083		0.090		0.137		0.067		0.083	
**title_length**													
	IRR	0.992		0.979^e^		1.018^f^		N/A^i^		N/A		N/A	
	SE	0.007		0.006		0.009		N/A		N/A		N/A	
**title_liwc_pos**													
	IRR	1.051		1.056		1.001		N/A		N/A		N/A	
	SE	0.124		0.112		0.077		N/A		N/A		N/A	
**title_liwc_neg**													
	IRR	0.914		1.083		1.007		N/A		N/A		N/A	
	SE	0.082		0.092		0.077		N/A		N/A		N/A	
**log_cov_tweet**													
	IRR	0.861		0.904		1.201		0.636^f^		0.842		0.874	
	SE	0.143		0.218		0.299		0.142		0.178		0.213	
**log_cov_case**													
	IRR	0.864		0.778^g^		0.846		0.717^g^		0.762^f^		0.728^f^	
	SE	0.155		0.100		0.146		0.144		0.095		0.091	
**log_cov_fatality**													
	IRR	1.144		1.254^f^		1.149		0.977		1.127		1.075	
	SE	0.178		0.136		0.175		0.168		0.119		0.131	
**ln(alpha)**													
	IRR	4.596^e^		4.369^e^		4.172^e^		3.593^e^		3.711^e^		3.367^e^	
	SE	0.151		0.120		0.143		0.165		0.119		0.113	
**constant**													
	IRR	0.188		0.089		0.000^f^		N/A		N/A		N/A	
	SE	0.503		0.337		0.002		N/A		N/A		N/A	

^a^Dependent variable: retweets in the first 168 hours.

^b^No fixed effect.

^c^Fixed effect.

^d^IRR: incidence rate ratio.

^e^*P*<.01.

^f^*P*<.05.

^g^*P*<.10.

^h^Robust standard error clustered by article.

^i^N/A: not applicable.

### Specific Emotion

Next, we examined the impact of a specific emotion on retweet count. In this analysis, we used a machine learning approach that was developed for tweet text analysis [51] rather than a general word count–based method. The algorithm classified the emotion in each tweet into 4 categories: joy (happiness), anger, fear, and sadness, as well as a neutral (no specific emotion) condition. For analytical purpose, we focused on these 4 basic emotions as they are the most commonly studied ones in the computational and evolutionary models of emotion [58,59]. Among the classified emotions of the combined tweet sample, 24.5% (59,674/243,567) involved joy, 5.0% (12,178/243,567) involved anger, 41.0% (99,862/243,567) involved fear, and 2.6% (6,333/243,576) involved sadness. This left 26.9% (65,520/243,567) of tweets that had no specific emotion. We have further provided details on the distribution of these specific emotions in all 3 subgroups in Table 3. The results of this classification were largely consistent with the findings of recent studies that have profiled public emotions on social media during the COVID-19 pandemic [10,40], where the authors also found a prevalence of negative emotions such as fear.

The regression analysis on the combined sample (see Multimedia Appendix 6) revealed that, compared with the presence of no specific emotion, joy was associated with a 25.6% increase in retweet count (IRR 1.256, 95% CI 1.158-1.362; *P<*.001), anger was associated with a 20.4% decrease in retweet count (IRR 0.796, 95% CI 0.702-0.901; *P<*.001), and both fear (IRR 0.998, 95% CI 0.908-1.097; *P=*.97) and sadness (IRR 0.946, 95% CI 0.723-1.237; *P=*.68) had no effect on retweet count. These results confirmed the general existence of a “positivity bias,” and only the positive emotion of joy contributed to content sharing. More importantly, high-arousal negative emotions, such as anger and fear, were found to have either a negative or neutral impact on content sharing.

**Figure 3:**
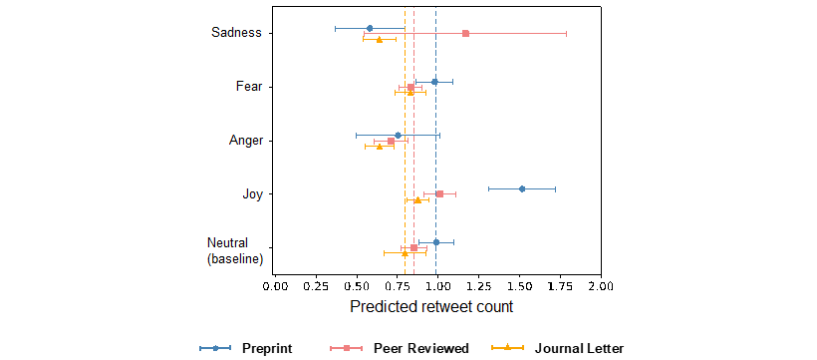
Prediction of retweet count according to emotion. Joy in tweets about preprints predicts the highest retweet count. Error bars indicate 95% CIs.

With further analysis, we again observed that the “positivity bias” was most prevalent in tweets mentioning preprints. In the combined sample (see Multimedia Appendix 6), the analysis revealed that the interaction between the preprint subgroup indicator and the joy indicator was significantly positive (IRR 1.290, 95% CI 1.092-1.524; *P=*.003). The interaction between the preprint subgroup indicator and the sadness indicator was significantly negative (IRR 0.429, 95% CI 0.334-0.524; *P=*.009). This difference was also observed in subgroup analysis (see Figure 3 and Models 1-3 in Table 5). More specifically, in the preprint subgroup, joy predicted a 50.3% increase in retweet count (IRR 1.503, 95% CI 1.324-1.707; *P<*.001) and sadness predicted a 41.0% decrease in retweet count (IRR 0.590, 95% CI 0.417-0.834; *P=*.003). Both high-arousal negative emotions (anger and fear) had neutral impacts on retweet count. In comparison, joy had a smaller but significant positive impact on retweet count (IRR 1.186, 95% CI 1.073-1.310; *P=*.001) in the journal research article subgroup but not in the opinion/letter subgroup. Similarly, anger was associated with less retweets (IRR 0.843, 95% CI 0.725-0.980; *P=*.03) in the journal research article subgroup but not in the opinion/letter subgroup. Sadness had negative effects on retweet count (IRR 0.810, 95% CI 0.671-0.977; *P=*.03) in the journal opinion/letter subgroup but not in the journal research article subgroup. Lastly, fear did not appear to have any effects across all subgroups. Additional results from fixed effect analysis of the within-article effects were again largely consistent (see Models 4-6 in Table 5). Thus, overall, our results showed that a positive-valence emotion, rather than a negative-valence emotion or high-arousal emotion, contributes to higher content sharing of social media messages about COVID-19 scientific research.

**Table 5:** Negative binomial estimation results using a specific emotion in subgroups.

Variable		Model 1 (preprint; N=47,570)^a,b^	Model 2 (peer reviewed; N=97,769)^a,b^	Model 3 (journal letter; N=98,228)^a,b^	Model 4 (preprint; N=47,570)^a,c^	Model 5 (peer reviewed; N=97,769)^a,c^	Model 6 (journal letter; N=98,228)^a,c^
**joy**							
	IRR^d^	1.503^e^	1.186^e^	1.100	1.317^e^	1.117^f^	1.110^g^
	SE^h^	0.098	0.060	0.086	0.094	0.056	0.061
**anger**							
	IRR	0.777	0.843^f^	0.835	0.809^f^	0.883	0.968
	SE	0.140	0.065	0.095	0.067	0.067	0.103
**fear**							
	IRR	0.998	0.985	1.023	1.152	0.971	0.992
	SE	0.079	0.056	0.106	0.109	0.055	0.064
**sadness**							
	IRR	0.590^e^	1.440	0.810^f^	0.619^e^	1.128	0.905
	SE	0.104	0.393	0.078	0.091	0.196	0.084
**log_follower**							
	IRR	1.786^e^	1.874^e^	1.893^e^	1.929^e^	1.914^e^	1.935^e^
	SE	0.059	0.024	0.026	0.025	0.022	0.024
**verified**							
	IRR	2.149^e^	1.892^e^	1.485^e^	2.029^e^	2.030^e^	1.821^e^
	SE	0.315	0.222	0.212	0.203	0.208	0.273
**length**							
	IRR	1.053^e^	1.052^e^	1.052^e^	1.056^e^	1.055^e^	1.050^e^
	SE	0.003	0.002	0.004	0.003	0.002	0.002
**hashtags**							
	IRR	1.037^f^	1.038^e^	1.010	1.063^e^	1.032^e^	1.017
	SE	0.017	0.013	0.017	0.014	0.011	0.018
**mention**							
	IRR	1.849^e^	1.593^e^	1.689^e^	1.596^e^	1.466^e^	1.618^e^
	SE	0.136	0.084	0.086	0.121	0.067	0.082
**title_length**							
	IRR	0.994	0.979^e^	1.015^g^	N/A^i^	N/A	N/A
	SE	0.006	0.006	0.009	N/A	N/A	N/A
**title_liwc_pos**							
	IRR	1.090	1.064	1.029	N/A	N/A	N/A
	SE	0.141	0.111	0.075	N/A	N/A	N/A
**title_liwc_neg**							
	IRR	0.934	1.096	1.047	N/A	N/A	N/A
	SE	0.077	0.099	0.072	N/A	N/A	N/A
**log_cov_tweet**							
	IRR	0.867	0.864	1.216	0.639^f^	0.845	0.879
	SE	0.142	0.176	0.309	0.143	0.177	0.215
**log_cov_case**							
	IRR	0.851	0.791^g^	0.853	0.723	0.759^f^	0.728^f^
	SE	0.152	0.100	0.142	0.145	0.094	0.091
**log_cov_fatality**							
	IRR	1.155	1.238^f^	1.144	0.970	1.132	1.075
	SE	0.179	0.133	0.169	0.166	0.119	0.131
**ln(alpha)**							
	IRR	4.536^e^	4.356^e^	4.167^e^	3.574^e^	3.708^e^	3.366^e^
	SE	0.158	0.116	0.142	0.164	0.118	0.113
**constant**							
	IRR	0.157	0.160	0.000^f^	N/A	N/A	N/A
	SE	0.417	0.519	0.001	N/A	N/A	N/A

^a^Dependent variable: retweets in the first 168 hours.

^b^No fixed effect.

^c^Fixed effect.

^d^IRR: incidence rate ratio.

^e^*P*<.01.

^f^*P*<.05.

^g^*P*<.10.

^h^Robust standard error clustered by article.

^i^N/A: not applicable.

### Role of Scientists as Social Media Message Creators

**Figure 4:**
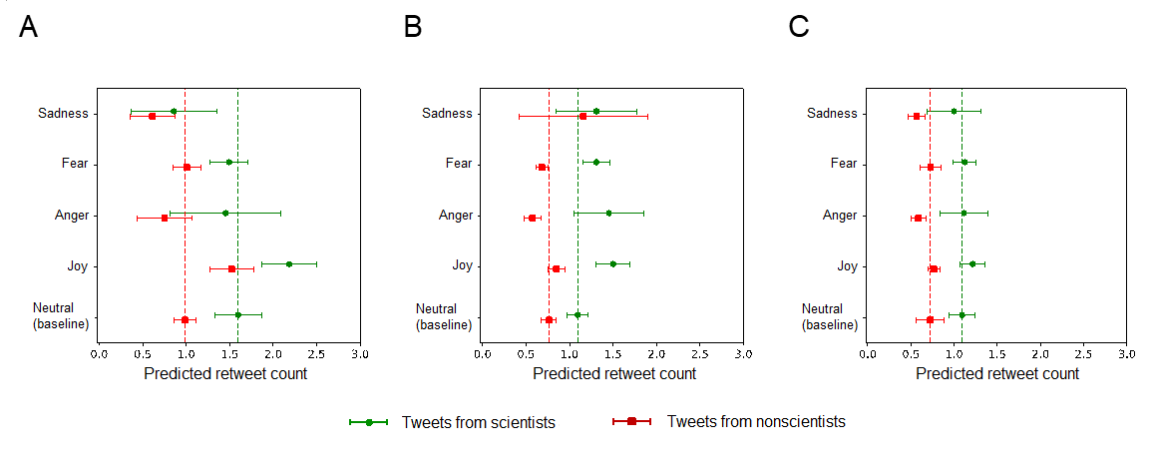
The retweet rate difference between tweets from scientists and nonscientists for (A) preprints, (B) peer-reviewed articles, and (C) journal letters. Error bars indicate 95% CIs.

We compared the difference in the retweet rate between tweets from scientists and nonscientists. The distributional differences of specific emotions between scientists and nonscientists in each subgroup are reported in Multimedia Appendix 7. In all subgroups (see Models 1-3 in Table 6), we observed a baseline “toning up” effect of scientists’ participation, where their tweets were associated with, on average, a 40%-60% higher retweet count than tweets from nonscientists (preprint: IRR 1.618, 95% CI 1.358-1.928; *P<*.001; journal research article: IRR 1.434, 95% CI 1.260-1.632; *P<*.001; journal opinion/letter pieces: IRR 1.513, 95% CI 1.204-1.901; *P<*.001). However, we only observed significant interaction effects between the scientist indicator and the emotion indicators for joy (IRR 1.235, 95% CI 1.031-1.479; *P=*.02), anger (IRR 1.767, 95% CI 1.262-2.474; *P=*.001), and fear (IRR 1.339, 95% CI 1.124-1.594; *P=*.001) in the journal research article subgroup. All other interaction terms were not significant (Figure 4). Further within-article effect analysis using fixed effects revealed consistent results (see Models 4-6 in Table 6).

These results highlighted that scientists’ participation could alter the emotional dynamics in the social media sharing of messages of preprints, as their expressed positive emotions (ie, joy) and high-arousal negative emotions (ie, anger and fear) could enhance sharing. In comparison, the indifferences in the emotional dynamics between scientists’ tweets and nonscientists’ tweets about preprints may suggest that it is the emotion elicited by the messages about preprints, rather than who expressed it, that influences content sharing.

**Table 6:** Negative binomial estimation results using interactions between the scientist indicator and the specific emotion indicators in subgroups.

Variable			Model 1 (preprint; N=47,570)^a,b^		Model 2 (peer reviewed; N=97,769)^a,b^		Model 3 (journal letter; N=98,228)^a,b^		Model 4 (preprint; N=47,570)^a,c^		Model 5 (peer reviewed; N=97,769)^a,c^		Model 6 (journal letter; N=98,228)^a,c^
**scientist**													
	IRR^d^	1.618^e^		1.434^e^		1.513^e^		1.667^e^		1.393^e^		1.601^e^	
	SE^f^	0.145		0.095		0.176		0.143		0.085		0.134	
**joy**													
	IRR	1.540^e^		1.110^g^		1.060		1.344^e^		1.044		1.102	
	SE	0.123		0.068		0.112		0.113		0.060		0.074	
**anger**													
	IRR	0.762		0.751^e^		0.811		0.819^h^		0.772^e^		0.975	
	SE	0.154		0.063		0.116		0.079		0.060		0.129	
**fear**													
	IRR	1.020		0.893^g^		1.008		1.194		0.890^g^		0.984	
	SE	0.097		0.059		0.140		0.130		0.057		0.080	
**sadness**													
	IRR	0.618^h^		1.516		0.783^h^		0.623^e^		1.057		0.883	
	SE	0.126		0.490		0.085		0.107		0.224		0.092	
**scientist × joy**													
	IRR	0.887		1.235^h^		1.048		0.914		1.236^h^		0.971	
	SE	0.119		0.114		0.139		0.116		0.106		0.099	
**scientist × anger**													
	IRR	1.194		1.767^e^		1.258		1.106		1.913^e^		1.034	
	SE	0.333		0.304		0.222		0.231		0.369		0.150	
**scientist × fear**													
	IRR	0.916		1.339^e^		1.019		0.875		1.330^e^		1.000	
	SE	0.105		0.119		0.151		0.103		0.101		0.096	
**scientist × sadness**													
	IRR	0.869		0.789		1.168		1.127		1.372		1.137	
	SE	0.317		0.281		0.213		0.376		0.374		0.187	
**log_follower**													
	IRR	1.772^e^		1.858^e^		1.878^e^		1.914^e^		1.895^e^		1.922^e^	
	SE	0.058		0.024		0.026		0.026		0.022		0.024	
**verified**													
	IRR	2.091^e^		1.733^e^		1.482^h^		1.986^e^		1.929^e^		1.800^e^	
	SE	0.315		0.210		0.228		0.207		0.207		0.284	
**length**													
	IRR	1.052^e^		1.052^e^		1.053^e^		1.055^e^		1.055^e^		1.050^e^	
	SE	0.004		0.002		0.004		0.003		0.002		0.002	
**hashtags**													
	IRR	1.038^h^		1.035^e^		1.011		1.066^e^		1.030^e^		1.020	
	SE	0.017		0.012		0.017		0.015		0.011		0.018	
**mention**													
	IRR	1.847^e^		1.560^e^		1.643^e^		1.602^e^		1.447^e^		1.599^e^	
	SE	0.140		0.082		0.086		0.125		0.066		0.081	
**title_length**													
	IRR	0.993		0.978^e^		1.011		N/A^i^		N/A		N/A	
	SE	0.006		0.006		0.008		N/A		N/A		N/A	
**title_liwc_pos**													
	IRR	1.073		1.026		1.026		N/A		N/A		N/A	
	SE	0.142		0.097		0.074		N/A		N/A		N/A	
**title_liwc_neg**													
	IRR	0.922		1.103		1.034		N/A		N/A		N/A	
	SE	0.075		0.097		0.071		N/A		N/A		N/A	
**log_cov_tweet**													
	IRR	0.863		0.878		1.207		0.636^h^		0.895		0.898	
	SE	0.140		0.176		0.309		0.145		0.191		0.225	
**log_cov_case**													
	IRR	0.848		0.826		0.863		0.719^g^		0.759^h^		0.740^h^	
	SE	0.153		0.106		0.147		0.144		0.097		0.091	
**log_cov_fatality**													
	IRR	1.151		1.187		1.131		0.977		1.140		1.074	
	SE	0.180		0.127		0.171		0.167		0.123		0.129	
**ln(alpha)**													
	IRR	4.489^e^		4.250^e^		4.098^e^		3.539^e^		3.624^e^		3.308^e^	
	SE	0.161		0.116		0.149		0.166		0.116		0.116	
**constant**													
	IRR	0.178		0.110		0.000^h^		N/A		N/A		N/A	
	SE	0.466		0.353		0.002		N/A		N/A		N/A	

^a^Dependent variable: retweets in the first 168 hours.

^b^No fixed effect.

^c^Fixed effect.

^d^IRR: incidence rate ratio.

^e^*P*<.01.

^f^Robust standard error clustered by article.

^g^*P*<.10.

^h^*P*<.05.

^i^N/A: not applicable.

## Discussion

### Principal Findings

The COVID-19 crisis may have already created a lasting change to the scientific communication process [60], leading this process to become more immediate and transparent as exemplified by explosive use and sharing of preprints. Should we be worried? Using 243,567 original tweets, which generated 729,319 retweets, about 8612 COVID-19 articles from medical peer-reviewed journals and preprint servers in the early months of the pandemic, we shed light on this question by investigating the emotional dynamics of social media sharing of COVID-19 scientific outputs. Our quantitative analyses revealed 3 key findings.

First, we observed a positivity bias. A positive-valence emotion, rather than a negative-valence emotion or high-arousal emotion, contributed to the sharing. Even though the pandemic has given COVID-19 research a heightened “news-like” status, the dissemination of this research on social media did not exhibit a pattern mimicking social media news. Instead, it implied that social media users’ sharing of COVID-19 science may be motivated by altruistic reasons or self-enhancement, which was consistent with previous studies on the sharing of science to the public in interpersonal communication settings [8]. However, to the best of our knowledge, the observed differential emotional dynamics of content sharing in messages that mentioned different sources (ie, preprints, peer-reviewed journal research, and journal opinion/letter pieces) have not been demonstrated previously.

Second, the “positivity bias” was most salient in messages of preprints than messages of articles in peer-reviewed journals. What drives this observed difference in emotional dynamics, especially between tweets about preprints and peer-reviewed research? One possibility could be the nature of preprints, as preprints involve nonvetted findings. The peer-review process helps scrutinize and mitigate the scientific uncertainty of a scientific manuscript, and the process often leads to tone-downed findings and conclusions [61]. Without undergoing this “toning down” process, the raw findings in preprints are more likely to be novel, eye-catching, and political [62], which could boost the effect of emotion on content sharing.

Given the self-enhancement explanation behind the “positivity bias,” it is also possible that tweets about preprints possess higher self-enhancement potential. Findings in preprints may be perceived by social media users to have higher self-enhancement value because they may be perceived as more novel and impactful [62]. Our explorative analysis using word cloud visualization could provide support for this conjecture as it implied that the positive language in tweets about preprints tends to contain more action-oriented positive words than tweets about peer-reviewed articles. This potential action-positivity perspective also aligns with a self-enhancement explanation, as self-enhancement is linked to not only a positive mindset and stress resistance, but also action orientation [63]. Future research efforts could expand on this conjecture to conduct more in-depth investigations.

Finally, we showed that scientists’ participation in the social media sharing of COVID-19 science exhibited differential emotional dynamics in tweets about different scientific sources. Specifically, scientists played a moderating role in the sharing of social media messages about peer-reviewed research, as their expressive positive emotions (ie, joy) and high-arousal negative emotions (ie, anger and fear) further enhanced sharing. However, the same pattern was not observed in messages about preprints. Given that peer-reviewed journal research contains arguably much more reliable findings than preprints, the presence of enhancing and neutral effects of scientists’ emotions in tweets about peer-reviewed research and preprints, respectively, could imply a moderated emotional communication process by scientists on social media, selectively promoting more reliable findings. Therefore, our study highlights the instrumental role of scientists in moderating science communication to the public on social media, echoing recent calls for promoting more effective science communication from both the scientific community [64] and the public [65] during crises.

### Limitations

Our focus on studying the messages that explicitly referenced COVID-19 research (ie, with a valid URL reference), however, limited us from examining other messages that may have contained scientific research information but did not provide a valid reference. Lack of a valid reference or source ambiguity is a key factor leading to rumor mongering [66] or differentiating science from science conspiracy on social media [67]. Examining the emotional dynamics in these types of messages would be an interesting future research direction. Would the “positivity bias” still exist or would a “negativity bias” prevail instead? Examining these questions would provide insights on social media management, especially the importance of a valid source reference in online messaging. Further, our study design could not fully explicate the causal relationship between the emotion present in tweet text and the subsequent diffusion (retweet). Studies that aim to examine such a causal relationship may consider a randomized study design using either a laboratory experiment or a large-scale field experiment. A future study could also expand on our study to examine the social media sharing of a broader range of scientific outputs beyond COVID-19. Additionally, we detected and translated non-English tweets using only the Google Translate API. Future studies may consider cross-validating this process with human verification or alternative approaches.

### Conclusions

Notwithstanding these limitations, our study provides useful implications that add to the ongoing debate regarding the virtue and danger in the use of preprints in science communication to the public [44-46]. Distorted social media dissemination of science could potentially resemble that of misinformation or scientific conspiracy. For instance, in a direct comparison of the online spread of scientific and conspiracy-theory content, a recent study showed that a negative emotion was more likely to enhance the engagement and virality of conspiracy content [30]. We provided evidence that, at least from the perspective of emotional dynamics, social media sharing of COVID-19 science did not exhibit such a distorted pattern that overtly promotes negative emotional messages. On the contrary, positive emotional messages were found to transmit faster, especially in preprints. However, the extent to which such positive but unverified findings of preprints are widely shared on social media was beyond the scope of this study. Practically, our findings highlighted the instrumental role played by scientists in promoting the dissemination of more reliable findings, which can have important implications for social media platform governance in terms of public discourse, especially during crises. Scientists could infuse messages about peer-reviewed articles with positive and high-arousal emotions but try to tone down the emotionality of messages about preprints to reduce the scientific uncertainty in communication. Scientists’ strategic use of emotions in social media sharing could help promote organized and orderly social media sharing of science without relying on explicit and centralized controls on the accessibility of preprints to the public.

## Appendix

Multimedia Appendix 1 Information on data processing and cleaning.

Multimedia Appendix 2 Information on the method for classifying scientist Twitter users.

Multimedia Appendix 3 Information on robustness tests.

Multimedia Appendix 4 Negative binomial estimation results using Linguistic Inquiry and Word Count emotional word counts in the combined sample.

Multimedia Appendix 5 Top 15 positive or negative emotion word stems (Linguistic Inquiry and Word Count 2015) in each text corpus.

Multimedia Appendix 6 Negative binomial estimation results using specific emotion indicators in the combined sample.

Multimedia Appendix 7 Kolmogorov-Smirnov test statistics on the distribution of specific emotions between tweets from scientists and tweets from nonscientists in each subgroup.

## Abbreviations

API: application programming interface
DOI: digital object identifier
IRR: incidence rate ratio
LIWC: Linguistic Inquiry and Word Count
