# Supplementary material for: The Asymmetric Influence of Emotion in the Sharing of COVID-19 Science on Social Media: Observational Study
Source: JMIR Infodemiology. 2022 Dec 8;2(2):e37331. doi: 10.2196/37331 (PMC9749104; doi:10.2196/37331)
Supplement: Multimedia Appendix 1 [file infodemiology_v2i2e37331_app1.docx]

**Multimedia Appendix 1.** Information on data processing and cleaning.

In this section, we describe in more details how we cleaned the data. Our original collection of COVID-19 research from PubMed returned 6,552 unique DOIs of English articles included in the MEDLINE database. We then used PubMed API to query its database to retrieve the article meta-data. The PubMed meta-data allowed to identify whether the article is an opinion/letter piece or research article, which yields 3,567 opinion/letter pieces and 2,985 research articles. Meanwhile, there were 3,725 preprints (which also had a unique DOI for each preprint) published in either bioRxiv or medRxiv. Figure S1 depicts the cumulative production of preprint, peer-reviewed research, opinion/letter pieces over the early months of COVID-19 pandemic.

We then queried the Altmetric database using the DOIs to retrieve all tweet mentions (tweet status id only). After hydrating the tweet using the status id using Twitter REST API, we had in total 629,900 raw tweets that are either original tweets, quoted tweets, or replies. Among them, 207,329 are replies and 154,568 are quoted tweets. A quoted tweet is a retweet of an original tweet with added text comment. A reply is a text replied to an original tweet. Although quoted tweets and replies can be retweeted separately, they are generally much less likely to be retweeted than original tweets. To remain focused, we used only the original tweets which left us an initial sample of 268,003 original tweets. Among this initial sample, we further proceed to remove any potential tweet from a non-human account. A non-human account is a Twitter account maintained by bot or an organization account (for example, PNAS maintains an organizational account *@PNASnews*, Science maintains an organizational account *@ScienceMagazine*, preprint server like medRxiv *@medrxivpreprint*). To identify the organizational account of those journal or preprints, we manually searched and matched on Twitter for all journals and preprints. Removing any tweets from these accounts removed 6,402 original tweets. Furthermore, we checked our data to identify the accounts with excessively large number of tweets (e.g., more than 200). We then manually visited and checked every identified account to remove any potential account operated by bot or non-publisher organizations. For example, the account *@COVID_evidence* is an automated bot account created in March 2020 to tweet about COVID-19 research. This account generated more than 2,000 original tweets in our data. The account *@outbreaksci* is an organizational account, created in 2016, for a non-profit to advance the science of outbreak response, which accounted for more than 8,000 original tweets in our data. Removing tweets from these accounts further eliminated 18,034 original tweets, yielding a final sample of 243,567 original tweets.

To prepare all text data for processing, we wrote a Python program to first detect the text language then translate it to English. The program used the *whatthelang* package to detect the language in the text, then translated the language into English using Google Translate Python package (*googletrans*). Afterward, the translated text was further processed to remove the URL, hashtags, and mentions to avoid the misinterpretation of those entity as text in later tasks of emotion measurement generation and user identity classification.

The control variables (see Tables 2 and 3 for detailed variable definition and summary statistics) used in this study include i) the (log-transformed) number of followers, ii) indicator of a verified user, iii) the number of the words in the tweet text, iv) number of hashtags used in the tweet, v) indicated of if there is any mention in the tweet, vi) the referenced article characteristics, vii) daily global COVID-19 situational variables. The first five variables are tweet-level controls can be directly generated from the raw tweet data retrieve by Twitter REST API. We did not include an indicator for URL presence simply due to that every tweet in our data contains a valid URL to the referenced article.

Besides the above tweet-level controls, we also control for the characteristics of the referenced article. More specifically, we considered the following three characteristics: i) the number of words in the title of the referenced article, ii) the number of positive emotion words in the title of referenced article, and iii) the number of negative emotion words in the title of referenced article. The count of positive and negative emotional words were generated through the same validated dictionaries (LIWC 2015 [49]) used in counting the emotional words in tweet texts.

To process COVID-19 situational variables. First, we transformed the daily global COVID-19 confirmed case and fatality data to a 7-day rolling total numbers. For example, if the focal date is March 10^th^, our rolling window would include the three days before and after March 10^th^ (i.e., from March 7^th^ to March 13^th^). Second, the daily global COVID-19 twitter data [48] contains only the tweet status ids in every hour starting from January 28^th^. Without hydrating the tens of millions of tweets, we simply counted the number of tweet status ids in each date. This leaves the dates before January 28^th^ as missing. We imputed the tweet count in the missing dates by implementing a linear prediction model using the first 30 days tweet counts. Similar to the COVID-19 case and fatality data, we transformed the daily global tweet count data to a 7-day rolling total numbers.

Lastly, to prepare the data for the wordcloud visualization shown in Figure 2 in the main text, we created four text corpuses along the emotion dimension (i.e., positive vs. negative) and tweet source dimension (i.e., preprint vs. peer-reviewed). For example, if a *positive* dictionary word identified using LIWC 2015 appears in a tweet or retweet text (the text in the retweet is exactly the text in the original tweet being retweeted) about a *preprint*, this word is added to the *positive-preprint* text corpus. Then, each word in the four text corpuses were then processed to keep only the word stem using the *PorterStemmer* in the Python *NLTK* package. Finally, we use another Python package *sklearn* to generate TF-IDF weight for each word in the text corpuses. The TF-IDF weight is what used to create the wordcloud.


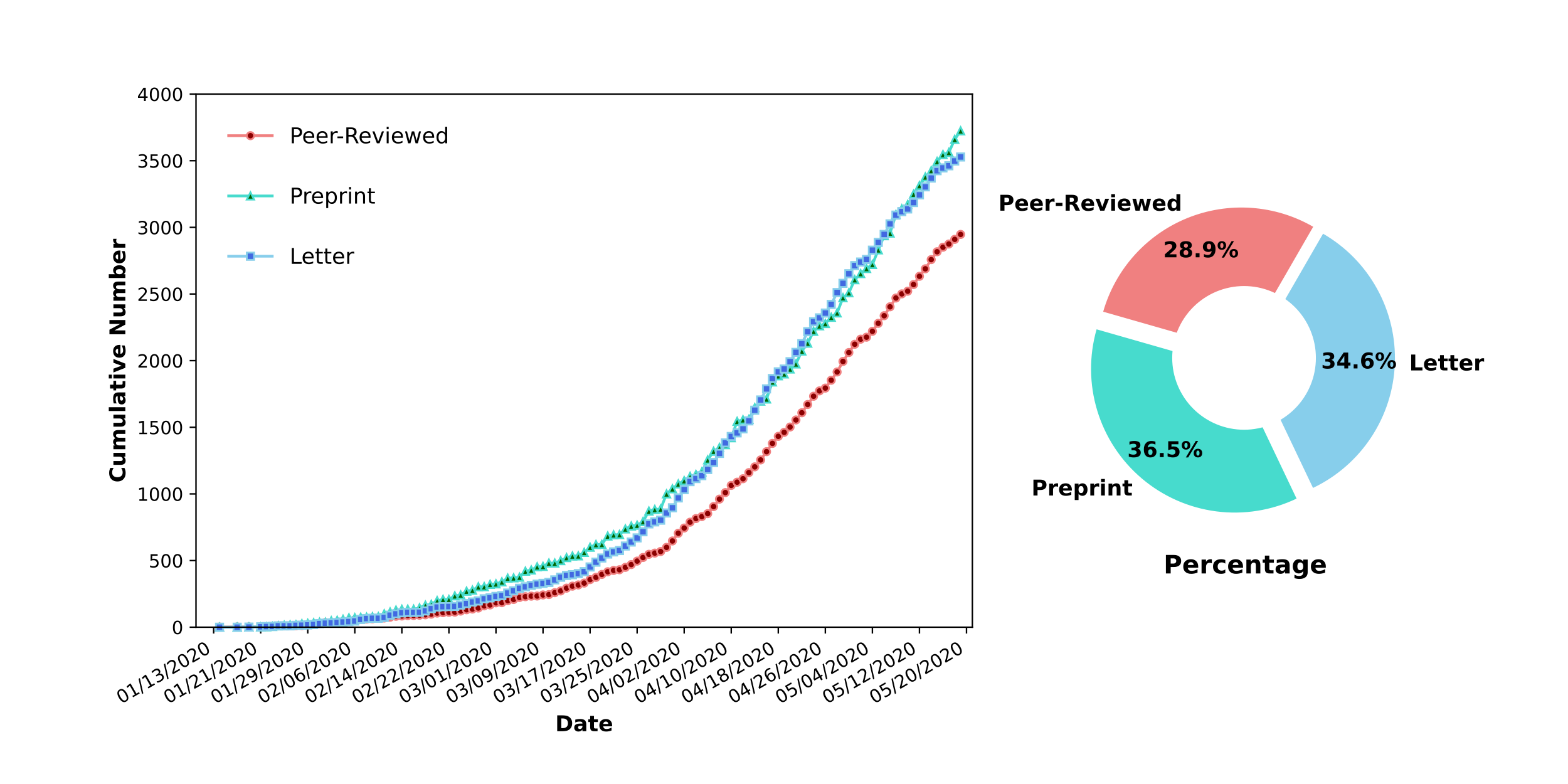
.0523815

**Figure S1**. The left-side chart depicts the cumulative production of preprint research articles, peer-reviewed research articles, journal opinion/letter pieces throughout our research period. The right-side pie chart shows the composition of articles in these three types at the end of our research period.
