# Supplementary material for: The Asymmetric Influence of Emotion in the Sharing of COVID-19 Science on Social Media: Observational Study
Source: JMIR Infodemiology. 2022 Dec 8;2(2):e37331. doi: 10.2196/37331 (PMC9749104; doi:10.2196/37331)
Supplement: Multimedia Appendix 2 [file infodemiology_v2i2e37331_app2.docx]

**Multimedia Appendix 2.** Information on the method for classifying scientist Twitter users.

Classifying Twitter users in healthcare domain has been a challenging task, a most recent study and review by Zhang and Bors [52] demonstrated that multi-class classification of a variety of healthcare stakeholders could only achieve a 55% classification F1 score. And many existing methods have been applied to a highly specific health domain [52], rather than a general context like COVID-19. As such, instead of applying some existing domain-specific classifiers, we use a two-step approach that relies on keyword identification and heuristic to identify scientist creators (i.e., doctors and academic researchers).

- **Step 1**: Tagging the words that indicating doctors, researchers, or scientists in all users’ self-reported biography description.
- **Step 2**: Using heuristic rules based on the tags generated in Step 1 to identify expert users.

To conduct Step 1, we adopt the approach of Ke, Ahn and Sugimoto [53] to curate a list of doctors, researcher, and scientist titles from three sources. We start with the 2018 Standard Occupational Classification (SOC) System^[[1]](#footnote-1)^ released by the Bureau of Labor Statistics, United States Department of Labor. More specifically, we used all occupations under the 19-1000 Life Scientists, 29-1020 Dentists, 29-1040 Optometrists, 29-1210 Physicians, and 29-1240 Surgeons subgroups. To ensure a more comprehensive coverage, we further consider two additional sources on the Internet to augment the list construction. First, we add all types of doctors from US News Doctors by Specialty page.^[[2]](#footnote-2)^ Second, we add all scientist title under Life Science from the Wikipedia page about scientist profession.^[[3]](#footnote-3)^ We combined the three lists together and removed duplicated entries.

Apart from the formal titles identified above, we also considered the informal and casual nature of social media. Thus, we also iteratively add additional words, such as acronym, to the list. We then use regular expression, a sequence of characters that define a search pattern^[[4]](#footnote-4)^, to check if a Twitter users’ bio contains any word from the list. Table below describes the full list of regular expression.

| **Regular Expression** | **Description** |
| --- | --- |
| (?i)\bKEYWORD\b | - Case insensitive matching of pattern “KEYWORD” from either the beginning or the end of a word - KEYWORD is a word from the list of titles, or in of the following:   - clinician   - doctor   - physician   - surgeon   - scientist   - researcher   - professor   - gyn   - ob-gyn   - ob/gyn |
| \bG\.P\.\b | Case insensitive matching of pattern “G.P.” from either the beginning or the end of a word |
| \bGP\b | Case insensitive matching of pattern “GP” from either the beginning or the end of a word |
| \bM\.D\.\b | Case insensitive matching of pattern “M.D.” from either the beginning or the end of a word |
| \bMD\b | Case insensitive matching of pattern “MD” from either the beginning or the end of a word |

The end results of step 1 are the creation of the <EXPERT> tag in Twitter user biography text. For example, if the biography contains the KEYWORD for expert (e.g., “*I am an* ***epidemiologist*** *working on COVID-19.”*), then step 1 outputs: “*I am an* ***<EXPERT>*** *working on COVID-19.*” If the biography contains no such KEYWORD (e.g., Mr. Trump’s biography: “*45th President of the United States of America*.”), then step 1 won’t assign any tag to the text. Building on the output of step 1, we then describe the heuristic rules we applied to identify expert user.

The key objective of Step 2 is to ensure that non-scientist Twitter users’ casual usage of medical or academic title in their biography does not result in us incorrectly identify them as experts. To facilitate achieving this goal, we first curate a list of medical specialties from Wikipedia^[[5]](#footnote-5)^. Similarly, we use this list to create the <SPECIALTY> tag in Twitter user biography. We then develop a set of heuristic rules based these two sets of tags and regular expression to classify a user as scientist or non-scientist. Table below describe the rules.

| **Rule** | **Description** | **Regular Expression** |
| --- | --- | --- |
| 1 | Using direct and unambiguous phrases to self-identify as <EXPRT> | - (?i) <EXPERT> PhD - (?i) <EXPERT> research\S - (?i) full\wtime <EXPERT> - (?i) certified <EXPERT> - (?i) registered <EXPERT> - (?i) clinical <EXPERT> - (?i) I('m\| am) a\w <EXPERT> - (?i) junior <EXPERT> - (?i) senior <EXPERT> - (?i) medical <EXPERT> - (?i)<EXPERT> at - (?i)<EXPERT> of - (?i)<EXPERT> work\S - <SPECIALTY> <EXPERT> - <EXPERT>( \w+)[2,12]<SPECIALTY> - (?i) <SPECIALTY> PhD - (?i) PhD in <SPECIALTY> - (?i) PhD of <SPECIALTY> |
| 2 | Self-identification as <EXPERT> using hashtag or “\|” | - ^#<EXPERT> - ^(#\w+ )[1,3]#<EXPERT> - \| <EXPERT> \| - \| <EXPERT> |
| 3 | Direct inclusion of “MD”, or “GP” at the end of users’ screen name | - MD$ - M\.D\.$ - GP$ - G.P.$ |

As the table shows, we develop three general categories of rules to classify scientist users. If the text patterns in a user’s biography or screen name satisfy any one of the rules described above, we consider this user as a scientist in our data. In essence, applying these rules ensures that the identification of scientist users is not solely based on the superficial spotting of keyword about medical or academic titles. The incorporation of additional information, being either a new attribute (e.g., SPECIALTY) or grammar, helps to provide the triangulation for a more accurate identification.

Validation Test:

To check the quality of the classification, we conduct validation test by comparing the classification results against the ground truth in smaller samples. The ground truth is constructed through manually labeling. More specially, we generated two random samples each of 200 users in our data. Of the around 1 million unique Twitter users in our data,^[[6]](#footnote-6)^ our classification algorithm predicted around 4.54% were scientist users. To facilitate a more reliable comparison, we used stratified random sampling based on the machine-predicted outcome to ensure that each of the samples contained exactly 10% of users who were predicted as scientists by the algorithm.

We then recruited and trained two independent research assistants (RAs) to manually classify these users into scientists and non-scientists. The RAs only received the links to each individual users’ Twitter page in the two samples without knowing classification outcomes. They needed to first inspect individual users’ biography and browse their tweet history. Then they decided using their best judgement whether the user is a medical doctor or researcher. The intercoder reliability measured by Cohen’s Kappa is 0.97. After this process was completed, we combined the manual classification results and labeled a user as scientist only if both RAs classified them as them as scientist. We then compared it against our automatedly generated labels. Below shows the classification confusion matrices on the two random samples.

|  | **Predicted /**  **Non-scientist** | **Predicted /**  **Scientist** |
| --- | --- | --- |
| **Random Sample 1 (n=200)** | | |
| **Labeled – Non-scientist** | 177 | 3 |
| **Labeled – Scientist** | 3 | 17 |
| **Random Sample 1 (n=200)** | | |
| **Labeled – Non-scientist** | 171 | 2 |
| **Labeled – Scientist** | 9 | 18 |

Overall, our classification algorithm achieved an average F1 score of 95.5% (Sample 1: 0.97; Sample 2: 0.94). Moreover, an average of 1.39% of the non-scientist users may be misclassified as scientists while 30% of scientist users may be misclassified as non-scientists. This validation results suggest that, when classifying a user as scientist, we are more likely to under-classify rather than over-classify.

1. <http://www.bls.gov/soc/> [↑](#footnote-ref-1)
2. <https://health.usnews.com/doctors/specialists-index> [↑](#footnote-ref-2)
3. <https://en.wikipedia.org/wiki/Scientist#By_field> [↑](#footnote-ref-3)
4. <https://en.wikipedia.org/wiki/Regular_expression> [↑](#footnote-ref-4)
5. <https://en.wikipedia.org/wiki/Medical_specialty> [↑](#footnote-ref-5)
6. This includes users of retweets. [↑](#footnote-ref-6)
