# Supplementary material for: The Asymmetric Influence of Emotion in the Sharing of COVID-19 Science on Social Media: Observational Study
Source: JMIR Infodemiology. 2022 Dec 8;2(2):e37331. doi: 10.2196/37331 (PMC9749104; doi:10.2196/37331)
Supplement: Multimedia Appendix 3 [file infodemiology_v2i2e37331_app3.docx]

**Multimedia Appendix 3.** Information on robustness tests.

In this section, we demonstrate that our finding under two general criteria i) alternative window for counting retweet and ii) excessive occurrence of zero in the retweet count.

In the first robustness tests, we used an alternative shorter window for counting the number of retweets received by each tweet. More specifically, we narrowed the window from a week (168 hours) to 2 days (48 hours). As reported in Table S1-S6, we showed that varying the temporal research window of counting the retweets did not change the interpretation of our key findings.

In the second robustness tests, we consider the potential problem of excessive zero in the dependent variables. In our data, around 67.2% of tweets received zero retweet in the first week. In this case, some researchers have argued that a zero-inflated model [56] that utilizes a zero-inflated probability distribution could provide a better fit to the data with frequent zero-valued observation. In essence, the zero-inflated count model is a modified count model which first considers a selection model (i.e., logit or probit) to account for the zero generating processes. Furthermore, our dependent variable still exhibit severe overdispersion even after all zero counts are removed (mean = 15.024, standard deviation = 149.433), we therefore used a zero-inflated negative binomial model [57] instead of the zero-inflated Poisson model. The zero-inflated negative binomial model is readily available in Stata 15.1 by simply using the “*zinb*” command. We specified a logit-based zero-inflation model using tweet-level characteristics [i.e., number of followers (*log_follower*), whether the tweeter is a verified user (*verified*), the length of the tweet text (*length*), the number of hashtags used in the tweet (*hashtags*), and whether the tweet contained any mention (*mention*)]. Again, all coefficients are highly similar to our original results in terms of both effect size and statistical significance.

**Table S1**. (Robustness – Alternative Retweet Count) negative binomial estimation results using LIWC emotional word count in subgroups. Coefficients are incidental rate ratio.

|  | (1) | (2) | (3) |
| --- | --- | --- | --- |
| **D.V.** | RT2D | | |
| **Subgroup** | *Preprint* | *Peer-reviewed* | *Journal Letter* |
|  |  |  |  |
| liwc_positive | 1.171*** | 1.049 | 1.047 |
|  | (0.046) | (0.031) | (0.050) |
| liwc_negative | 0.975 | 1.031 | 1.045 |
|  | (0.050) | (0.049) | (0.058) |
| log_follower | 1.798*** | 1.892*** | 1.907*** |
|  | (0.061) | (0.028) | (0.028) |
| verified | 2.012*** | 1.859*** | 1.441*** |
|  | (0.280) | (0.225) | (0.200) |
| length | 1.049*** | 1.049*** | 1.050*** |
|  | (0.004) | (0.002) | (0.004) |
| hashtags | 1.042** | 1.032** | 0.998 |
|  | (0.018) | (0.013) | (0.014) |
| mention | 1.971*** | 1.606*** | 1.712*** |
|  | (0.153) | (0.083) | (0.088) |
| title_length | 0.992 | 0.979*** | 1.018** |
|  | (0.007) | (0.006) | (0.009) |
| title_liwc_pos | 1.063 | 1.056 | 1.000 |
|  | (0.128) | (0.114) | (0.078) |
| title_liwc_neg | 0.916 | 1.082 | 0.998 |
|  | (0.082) | (0.091) | (0.076) |
| log_cov_tweet | 0.868 | 0.904 | 1.187 |
|  | (0.146) | (0.223) | (0.304) |
| log_cov_case | 0.845 | 0.790* | 0.845 |
|  | (0.152) | (0.099) | (0.147) |
| log_cov_fatality | 1.165 | 1.236** | 1.146 |
|  | (0.182) | (0.130) | (0.177) |
| ln(alpha) | 4.581*** | 4.382*** | 4.179*** |
|  | (0.158) | (0.123) | (0.146) |
| Constant | 0.161 | 0.078 | 0.001* |
|  | (0.440) | (0.299) | (0.002) |
|  |  |  |  |
| Observations | 47,570 | 97,769 | 98,228 |

Robust standard errors clustered by article in parentheses

*** p<0.01, ** p<0.05, * p<0.1

**Table S2.** (Robustness – Alternative Retweet Count) negative binomial estimation results using specific emotion indicators in subgroups. Coefficients are incidental rate ratio.

|  | (1) | (2) | (3) |
| --- | --- | --- | --- |
| **D.V.** | RT2D | | |
| **Subgroup** | *Preprint* | *Peer-reviewed* | *Journal Letter* |
|  |  |  |  |
| joy | 1.490*** | 1.174*** | 1.108 |
|  | (0.096) | (0.059) | (0.089) |
| anger | 0.749 | 0.862* | 0.853 |
|  | (0.136) | (0.067) | (0.099) |
| fear | 0.997 | 0.984 | 1.035 |
|  | (0.079) | (0.056) | (0.109) |
| sadness | 0.614*** | 1.426 | 0.801** |
|  | (0.110) | (0.395) | (0.077) |
| log_follower | 1.800*** | 1.887*** | 1.909*** |
|  | (0.061) | (0.025) | (0.028) |
| verified | 2.114*** | 1.885*** | 1.459*** |
|  | (0.311) | (0.223) | (0.209) |
| length | 1.052*** | 1.052*** | 1.052*** |
|  | (0.003) | (0.002) | (0.004) |
| hashtags | 1.037** | 1.033** | 0.996 |
|  | (0.017) | (0.013) | (0.014) |
| mention | 1.879*** | 1.595*** | 1.699*** |
|  | (0.141) | (0.084) | (0.085) |
| title_length | 0.994 | 0.979*** | 1.015* |
|  | (0.006) | (0.006) | (0.009) |
| title_liwc_pos | 1.104 | 1.065 | 1.030 |
|  | (0.146) | (0.114) | (0.076) |
| title_liwc_neg | 0.936 | 1.094 | 1.040 |
|  | (0.078) | (0.097) | (0.070) |
| log_cov_tweet | 0.870 | 0.864 | 1.202 |
|  | (0.144) | (0.180) | (0.313) |
| log_cov_case | 0.835 | 0.801* | 0.851 |
|  | (0.150) | (0.099) | (0.144) |
| log_cov_fatality | 1.173 | 1.222* | 1.142 |
|  | (0.182) | (0.128) | (0.171) |
| ln(alpha) | 4.520*** | 4.369*** | 4.174*** |
|  | (0.164) | (0.120) | (0.145) |
| Constant | 0.143 | 0.140 | 0.000** |
|  | (0.385) | (0.463) | (0.002) |
|  |  |  |  |
| Observations | 47,570 | 97,769 | 98,228 |

Robust standard errors clustered by article in parentheses

*** p<0.01, ** p<0.05, * p<0.1

**Table S3.** (Robustness – Alternative Retweet Count) negative binomial estimation results on the interactions between specific emotion indicators and scientist indicators in subgroups. Coefficients are incidental rate ratio.

|  | (1) | (2) | (3) |
| --- | --- | --- | --- |
| **D.V.** | RT2D | | |
| **Subgroup** | *Preprint* | *Peer-reviewed* | *Journal Letter* |
|  |  |  |  |
| scientist | 1.659*** | 1.421*** | 1.524*** |
|  | (0.148) | (0.095) | (0.181) |
| joy | 1.528*** | 1.106* | 1.071 |
|  | (0.120) | (0.067) | (0.116) |
| anger | 0.735 | 0.768*** | 0.831 |
|  | (0.149) | (0.065) | (0.120) |
| fear | 1.027 | 0.888* | 1.023 |
|  | (0.097) | (0.059) | (0.145) |
| sadness | 0.649** | 1.487 | 0.764** |
|  | (0.133) | (0.492) | (0.081) |
| scientist × joy | 0.887 | 1.209** | 1.037 |
|  | (0.118) | (0.109) | (0.139) |
| scientist × anger | 1.204 | 1.768*** | 1.238 |
|  | (0.332) | (0.304) | (0.218) |
| scientist × fear | 0.891 | 1.362*** | 1.004 |
|  | (0.102) | (0.123) | (0.151) |
| scientist × sadness | 0.835 | 0.826 | 1.220 |
|  | (0.313) | (0.303) | (0.218) |
| log_follower | 1.786*** | 1.871*** | 1.894*** |
|  | (0.060) | (0.025) | (0.027) |
| verified | 2.062*** | 1.727*** | 1.454** |
|  | (0.311) | (0.210) | (0.225) |
| length | 1.052*** | 1.051*** | 1.052*** |
|  | (0.004) | (0.002) | (0.004) |
| hashtags | 1.038** | 1.030** | 0.996 |
|  | (0.017) | (0.012) | (0.015) |
| mention | 1.879*** | 1.560*** | 1.653*** |
|  | (0.144) | (0.082) | (0.084) |
| title_length | 0.993 | 0.978*** | 1.011 |
|  | (0.006) | (0.006) | (0.008) |
| title_liwc_pos | 1.084 | 1.025 | 1.029 |
|  | (0.147) | (0.099) | (0.076) |
| title_liwc_neg | 0.925 | 1.098 | 1.027 |
|  | (0.076) | (0.094) | (0.070) |
| log_cov_tweet | 0.866 | 0.877 | 1.194 |
|  | (0.142) | (0.179) | (0.314) |
| log_cov_case | 0.832 | 0.833 | 0.861 |
|  | (0.150) | (0.106) | (0.149) |
| log_cov_fatality | 1.170 | 1.178 | 1.129 |
|  | (0.182) | (0.125) | (0.174) |
| ln(alpha) | 4.469*** | 4.263*** | 4.105*** |
|  | (0.168) | (0.120) | (0.153) |
| Constant | 0.162 | 0.100 | 0.000* |
|  | (0.431) | (0.328) | (0.002) |
|  |  |  |  |
| Observations | 47,570 | 97,769 | 98,228 |

Robust standard errors clustered by article in parentheses

*** p<0.01, ** p<0.05, * p<0.1

**Table S4**. (Robustness – Alternative Model) zero-inflated negative binomial estimation results using LIWC emotional word count in subgroups. Coefficients are incidental rate ratio.

|  | (1) | (2) | (3) |
| --- | --- | --- | --- |
| **D.V.** | RT7D | | |
| **Subgroup** | *Preprint* | *Peer-reviewed* | *Journal Letter* |
|  |  |  |  |
| liwc_positive | 1.168*** | 1.038 | 1.032 |
|  | (0.045) | (0.029) | (0.045) |
| liwc_negative | 0.979 | 1.034 | 1.041 |
|  | (0.053) | (0.048) | (0.055) |
| log_follower | 1.688*** | 1.786*** | 1.779*** |
|  | (0.066) | (0.038) | (0.032) |
| verified | 2.070*** | 1.875*** | 1.517*** |
|  | (0.279) | (0.224) | (0.203) |
| length | 1.040*** | 1.041*** | 1.050*** |
|  | (0.005) | (0.003) | (0.006) |
| hashtags | 0.967** | 0.971** | 0.958*** |
|  | (0.013) | (0.013) | (0.015) |
| mention | 1.996*** | 1.598*** | 1.560*** |
|  | (0.172) | (0.084) | (0.083) |
| title_length | 0.992 | 0.979*** | 1.017** |
|  | (0.006) | (0.006) | (0.008) |
| title_liwc_pos | 1.055 | 1.039 | 1.014 |
|  | (0.129) | (0.106) | (0.079) |
| title_liwc_neg | 0.907 | 1.092 | 1.006 |
|  | (0.079) | (0.087) | (0.075) |
| log_cov_tweet | 0.878 | 0.953 | 1.226 |
|  | (0.156) | (0.238) | (0.304) |
| log_cov_case | 0.836 | 0.774** | 0.843 |
|  | (0.153) | (0.100) | (0.139) |
| log_cov_fatality | 1.179 | 1.264** | 1.157 |
|  | (0.186) | (0.136) | (0.169) |
| Constant | 0.362 | 0.086 | 0.001* |
|  | (1.042) | (0.333) | (0.003) |
|  |  |  |  |
| Observations | 47,570 | 97,769 | 98,228 |

Robust standard errors clustered by article in parentheses

*** p<0.01, ** p<0.05, * p<0.1

**Table S5.** (Robustness – Alternative Model) zero-inflated negative binomial estimation results using specific emotion indicators in subgroups. Coefficients are incidental rate ratio.

|  | (1) | (2) | (3) |
| --- | --- | --- | --- |
| **D.V.** | RT7D | | |
| **Subgroup** | *Preprint* | *Peer-reviewed* | *Journal Letter* |
|  |  |  |  |
| joy | 1.424*** | 1.133** | 1.079 |
|  | (0.094) | (0.060) | (0.080) |
| anger | 0.764 | 0.820** | 0.848 |
|  | (0.139) | (0.066) | (0.094) |
| fear | 0.940 | 0.945 | 1.007 |
|  | (0.069) | (0.055) | (0.099) |
| sadness | 0.583*** | 1.484 | 0.791** |
|  | (0.104) | (0.445) | (0.077) |
| log_follower | 1.691*** | 1.778*** | 1.781*** |
|  | (0.067) | (0.034) | (0.033) |
| verified | 2.169*** | 1.901*** | 1.534*** |
|  | (0.307) | (0.219) | (0.211) |
| length | 1.044*** | 1.044*** | 1.051*** |
|  | (0.004) | (0.003) | (0.006) |
| hashtags | 0.964*** | 0.971** | 0.957*** |
|  | (0.013) | (0.013) | (0.015) |
| mention | 1.855*** | 1.581*** | 1.542*** |
|  | (0.154) | (0.086) | (0.080) |
| title_length | 0.994 | 0.980*** | 1.014* |
|  | (0.006) | (0.006) | (0.008) |
| title_liwc_pos | 1.095 | 1.045 | 1.034 |
|  | (0.148) | (0.106) | (0.077) |
| title_liwc_neg | 0.925 | 1.108 | 1.045 |
|  | (0.075) | (0.094) | (0.070) |
| log_cov_tweet | 0.877 | 0.905 | 1.243 |
|  | (0.155) | (0.187) | (0.313) |
| log_cov_case | 0.821 | 0.788* | 0.850 |
|  | (0.151) | (0.100) | (0.137) |
| log_cov_fatality | 1.193 | 1.245** | 1.152 |
|  | (0.189) | (0.132) | (0.165) |
| Constant | 0.365 | 0.175 | 0.001** |
|  | (1.045) | (0.573) | (0.002) |
|  |  |  |  |
| Observations | 47,570 | 97,769 | 98,228 |

Robust standard errors clustered by article in parentheses

*** p<0.01, ** p<0.05, * p<0.1

**Table S6.** (Robustness – Alternative Model) zero-inflated negative binomial estimation results on the interactions between specific emotion indicators and scientist indicators in subgroups. Coefficients are incidental rate ratio.

|  | (1) | (2) | (3) |
| --- | --- | --- | --- |
| **D.V.** | RT7D | | |
| **Subgroup** | *Preprint* | *Peer-reviewed* | *Journal Letter* |
|  |  |  |  |
| scientist | 1.542*** | 1.387*** | 1.470*** |
|  | (0.144) | (0.097) | (0.168) |
| joy | 1.474*** | 1.064 | 1.044 |
|  | (0.123) | (0.069) | (0.105) |
| anger | 0.750 | 0.726*** | 0.826 |
|  | (0.155) | (0.065) | (0.117) |
| fear | 0.962 | 0.852** | 0.992 |
|  | (0.087) | (0.058) | (0.132) |
| sadness | 0.614** | 1.578 | 0.764** |
|  | (0.129) | (0.560) | (0.084) |
| scientist × joy | 0.863 | 1.215** | 1.057 |
|  | (0.119) | (0.118) | (0.138) |
| scientist × anger | 1.165 | 1.719*** | 1.240 |
|  | (0.340) | (0.294) | (0.225) |
| scientist × fear | 0.924 | 1.357*** | 1.037 |
|  | (0.107) | (0.126) | (0.152) |
| scientist × sadness | 0.837 | 0.703 | 1.183 |
|  | (0.310) | (0.269) | (0.221) |
| log_follower | 1.682*** | 1.773*** | 1.772*** |
|  | (0.067) | (0.035) | (0.032) |
| verified | 2.117*** | 1.742*** | 1.536*** |
|  | (0.307) | (0.208) | (0.226) |
| length | 1.043*** | 1.043*** | 1.052*** |
|  | (0.005) | (0.003) | (0.006) |
| hashtags | 0.968** | 0.974** | 0.961** |
|  | (0.013) | (0.012) | (0.016) |
| mention | 1.853*** | 1.548*** | 1.505*** |
|  | (0.160) | (0.085) | (0.080) |
| title_length | 0.993 | 0.978*** | 1.010 |
|  | (0.006) | (0.006) | (0.008) |
| title_liwc_pos | 1.080 | 1.010 | 1.031 |
|  | (0.150) | (0.092) | (0.075) |
| title_liwc_neg | 0.915 | 1.113 | 1.035 |
|  | (0.073) | (0.093) | (0.070) |
| log_cov_tweet | 0.871 | 0.915 | 1.225 |
|  | (0.154) | (0.184) | (0.307) |
| log_cov_case | 0.818 | 0.820 | 0.863 |
|  | (0.153) | (0.106) | (0.142) |
| log_cov_fatality | 1.189 | 1.198* | 1.136 |
|  | (0.191) | (0.128) | (0.167) |
| Constant | 0.162 | 0.100 | 0.000* |
|  | (0.431) | (0.328) | (0.002) |
|  |  |  |  |
| Observations | 47,570 | 97,769 | 98,228 |

Robust standard errors clustered by article in parentheses

*** p<0.01, ** p<0.05, * p<0.1
