# Supplementary material for: The Asymmetric Influence of Emotion in the Sharing of COVID-19 Science on Social Media: Observational Study
Source: JMIR Infodemiology. 2022 Dec 8;2(2):e37331. doi: 10.2196/37331 (PMC9749104; doi:10.2196/37331)
Supplement: Multimedia Appendix 4 [file infodemiology_v2i2e37331_app4.docx]

**Multimedia Appendix 4.** Negative binomial estimation results using Linguistic Inquiry and Word Count emotional word counts in the combined sample.

Coefficients are incidental rate ratio.

|  | (1) | (2) | (3) |
| --- | --- | --- | --- |
| **D.V.** | RT7D | | |
|  |  |  |  |
| liwc_positive | 1.075*** | 1.072*** | 1.048* |
|  | (0.025) | (0.025) | (0.030) |
| liwc_negative | 1.015 | 1.025 | 1.041 |
|  | (0.033) | (0.034) | (0.049) |
| preprint |  | 1.257*** | 1.229*** |
|  |  | (0.062) | (0.064) |
| letter |  | 0.912* | 0.913* |
|  |  | (0.043) | (0.043) |
| preprint × liwc_positive |  |  | 1.129*** |
|  |  |  | (0.051) |
| letter × liwc_positive |  |  | 0.990 |
|  |  |  | (0.047) |
| preprint × liwc_negative |  |  | 0.907 |
|  |  |  | (0.063) |
| letter × liwc_negative |  |  | 1.011 |
|  |  |  | (0.077) |
| log_follower | 1.858*** | 1.860*** | 1.860*** |
|  | (0.020) | (0.020) | (0.020) |
| verified | 1.715*** | 1.712*** | 1.707*** |
|  | (0.131) | (0.133) | (0.132) |
| length | 1.051*** | 1.050*** | 1.050*** |
|  | (0.002) | (0.002) | (0.002) |
| hashtags | 1.029*** | 1.029*** | 1.029*** |
|  | (0.009) | (0.009) | (0.009) |
| mention | 1.699*** | 1.715*** | 1.715*** |
|  | (0.056) | (0.057) | (0.058) |
| title_length | 1.003 | 0.995 | 0.994 |
|  | (0.005) | (0.004) | (0.004) |
| title_liwc_pos | 0.999 | 1.036 | 1.044 |
|  | (0.057) | (0.059) | (0.060) |
| title_liwc_neg | 1.011 | 1.020 | 1.019 |
|  | (0.051) | (0.051) | (0.050) |
| log_cov_tweet | 0.983 | 1.018 | 1.014 |
|  | (0.152) | (0.159) | (0.157) |
| log_cov_case | 0.817** | 0.826** | 0.821** |
|  | (0.077) | (0.077) | (0.077) |
| log_cov_fatality | 1.197** | 1.184** | 1.190** |
|  | (0.097) | (0.095) | (0.096) |
| ln(alpha)^[[1]](#footnote-1)^ | 4.379*** | 4.359*** | 4.356*** |
|  | (0.080) | (0.081) | (0.081) |
| Constant | 0.017* | 0.010* | 0.011* |
|  | (0.040) | (0.024) | (0.026) |
|  |  |  |  |
| Observations | 243,567 | 243,567 | 243,567 |

Robust standard errors clustered by article in parentheses

*** p<0.01, ** p<0.05, * p<0.1

1. The coefficients in this row display the natural exponential of ln(*alpha*) which is *alpha* – the estimated dispersion parameter for the conditional mean and variance in the negative binomial model. The larger the *alpha*, the more severe the dispersion. An insignificant *alpha* (or ln(*alpha*)) would imply a failure to reject the null hypotheses that *alpha* equals 1, which would suggest no dispersion in the conditional mean and variance. [↑](#footnote-ref-1)
