# Supplementary material for: The Asymmetric Influence of Emotion in the Sharing of COVID-19 Science on Social Media: Observational Study
Source: JMIR Infodemiology. 2022 Dec 8;2(2):e37331. doi: 10.2196/37331 (PMC9749104; doi:10.2196/37331)
Supplement: Multimedia Appendix 5 [file infodemiology_v2i2e37331_app5.docx]

**Multimedia Appendix 5.** Top 15 positive or negative emotion word stems (Linguistic Inquiry and Word Count 2015) in each text corpus.

| **Corpus** | **Positive /**  **Preprint** | | **Positive /**  **Peer-Reviewed** | | **Negative /**  **Preprint** | | **Negative /**  **Peer-Reviewed** | |
| --- | --- | --- | --- | --- | --- | --- | --- | --- |
| Order | **Word Stem** | **TF-IDF** | **Word Stem** | **TF-IDF** | **Word Stem** | **TF-IDF** | **Word Stem** | **TF-IDF** |
| 1 | import | 0.496 | import | 0.555 | fatal | 0.495 | isol | 0.618 |
| 2 | posit | 0.434 | care | 0.479 | lower | 0.411 | inhibit | 0.369 |
| 3 | good | 0.309 | posit | 0.333 | isol | 0.396 | critic | 0.233 |
| 4 | **hope** | 0.242 | good | 0.276 | low | 0.335 | inhibitor | 0.210 |
| 5 | **support** | 0.220 | great | 0.183 | critic | 0.218 | low | 0.209 |
| 6 | great | 0.217 | improv | 0.181 | inhibitor | 0.180 | fatal | 0.188 |
| 7 | **promis** | 0.212 | benefit | 0.132 | danger | 0.148 | failur | 0.182 |
| 8 | use | 0.202 | approv | 0.127 | inhibit | 0.133 | lower | 0.165 |
| 9 | share | 0.187 | support | 0.121 | strain | 0.127 | kill | 0.155 |
| 10 | care | 0.180 | share | 0.114 | risk | 0.120 | seriou | 0.133 |
| 11 | better | 0.121 | better | 0.109 | damag | 0.117 | sick | 0.132 |
| 12 | strong | 0.114 | strongest | 0.105 | bad | 0.109 | danger | 0.125 |
| 13 | improv | 0.103 | prize | 0.091 | domin | 0.106 | aggrav | 0.110 |
| 14 | benefit | 0.100 | hope | 0.090 | failur | 0.105 | worsen | 0.106 |
| 15 | thank | 0.093 | best | 0.076 | poorli | 0.101 | risk | 0.104 |
