# Supplementary material for: The Asymmetric Influence of Emotion in the Sharing of COVID-19 Science on Social Media: Observational Study
Source: JMIR Infodemiology. 2022 Dec 8;2(2):e37331. doi: 10.2196/37331 (PMC9749104; doi:10.2196/37331)
Supplement: Multimedia Appendix 6 [file infodemiology_v2i2e37331_app6.docx]

**Multimedia Appendix 6.** Negative binomial estimation results using specific emotion indicators in the combined sample.

Coefficients are incidental rate ratio.

|  | (1) | (2) | (3) |
| --- | --- | --- | --- |
| **D.V.** | RT7D | | |
|  |  |  |  |
| joy | 1.256*** | 1.228*** | 1.188*** |
|  | (0.052) | (0.050) | (0.061) |
| anger | 0.796*** | 0.806*** | 0.831** |
|  | (0.051) | (0.053) | (0.063) |
| fear | 0.998 | 1.004 | 0.975 |
|  | (0.048) | (0.050) | (0.055) |
| sadness | 0.946 | 0.962 | 1.370 |
|  | (0.130) | (0.133) | (0.369) |
| preprint |  | 1.240*** | 1.160** |
|  |  | (0.060) | (0.078) |
| letter |  | 0.932 | 0.934 |
|  |  | (0.044) | (0.084) |
| preprint × joy |  |  | 1.290*** |
|  |  |  | (0.110) |
| letter × joy |  |  | 0.925 |
|  |  |  | (0.088) |
| preprint × anger |  |  | 0.917 |
|  |  |  | (0.167) |
| letter × anger |  |  | 0.966 |
|  |  |  | (0.123) |
| preprint × fear |  |  | 1.015 |
|  |  |  | (0.093) |
| letter × fear |  |  | 1.068 |
|  |  |  | (0.116) |
| preprint × sadness |  |  | 0.429*** |
|  |  |  | (0.138) |
| letter × sadness |  |  | 0.585* |
|  |  |  | (0.167) |
| log_follower | 1.860*** | 1.862*** | 1.859*** |
|  | (0.020) | (0.020) | (0.020) |
| verified | 1.735*** | 1.732*** | 1.747*** |
|  | (0.135) | (0.138) | (0.136) |
| length | 1.053*** | 1.052*** | 1.052*** |
|  | (0.002) | (0.002) | (0.002) |
| hashtags | 1.026*** | 1.027*** | 1.028*** |
|  | (0.009) | (0.009) | (0.009) |
| mention | 1.670*** | 1.688*** | 1.685*** |
|  | (0.054) | (0.056) | (0.055) |
| title_length | 1.001 | 0.994 | 0.994 |
|  | (0.005) | (0.004) | (0.004) |
| title_liwc_pos | 1.028 | 1.061 | 1.065 |
|  | (0.057) | (0.059) | (0.059) |
| title_liwc_neg | 1.029 | 1.041 | 1.043 |
|  | (0.051) | (0.051) | (0.051) |
| log_cov_tweet | 1.001 | 1.030 | 1.000 |
|  | (0.156) | (0.162) | (0.147) |
| log_cov_case | 0.820** | 0.828** | 0.827** |
|  | (0.077) | (0.076) | (0.076) |
| log_cov_fatality | 1.191** | 1.180** | 1.182** |
|  | (0.096) | (0.094) | (0.093) |
| ln(alpha) | 4.361*** | 4.345*** | 4.334*** |
|  | (0.080) | (0.081) | (0.080) |
| Constant | 0.012* | 0.008** | 0.012* |
|  | (0.029) | (0.019) | (0.029) |
|  |  |  |  |
| Observations | 243,567 | 243,567 | 243,567 |

Robust standard errors clustered by article in parentheses

*** p<0.01, ** p<0.05, * p<0.1
