# Supplementary material for: The Asymmetric Influence of Emotion in the Sharing of COVID-19 Science on Social Media: Observational Study
Source: JMIR Infodemiology. 2022 Dec 8;2(2):e37331. doi: 10.2196/37331 (PMC9749104; doi:10.2196/37331)
Supplement: Multimedia Appendix 7 [file infodemiology_v2i2e37331_app7.docx]

**Multimedia Appendix 7.** Kolmogorov-Smirnov test statistics on the distribution of specific emotions between tweets from scientists and tweets from nonscientists in each subgroup.

| **Emotion** | ***Tweets from scientists*** | | ***Tweets from non-scientists*** | | **K-S Test** | **p-value** |
| --- | --- | --- | --- | --- | --- | --- |
|  | ***Mean*** | ***S.D.*** | ***Mean*** | ***S.D.*** |  |  |
| ***Preprint*** | | | | | | |
| joy | 0.286 | 0.452 | 0.279 | 0.449 | D=0.006 | 0.959 |
| anger | 0.031 | 0.174 | 0.047 | 0.212 | D=0.016 | 0.083 |
| fear | 0.428 | 0.495 | 0.395 | 0.489 | D=0.033 | <0.001 |
| sadness | 0.012 | 0.110 | 0.022 | 0.148 | D=0.010 | 0.531 |
| neutral | 0.242 | 0.429 | 0.256 | 0.436 | D=0.013 | 0.212 |
| ***Peer-reviewed*** | | | | | | |
| joy | 0.255 | 0.436 | 0.247 | 0.431 | D=0.008 | 0.288 |
| anger | 0.022 | 0.148 | 0.036 | 0.187 | D=0.014 | 0.008 |
| fear | 0.456 | 0.498 | 0.407 | 0.491 | D=0.050 | <0.001 |
| sadness | 0.016 | 0.127 | 0.023 | 0.148 | D=0.006 | 0.659 |
| neutral | 0.250 | 0.433 | 0.288 | 0.453 | D=0.038 | <0.001 |
| ***Journal Letter*** | | | | | | |
| joy | 0.260 | 0.439 | 0.215 | 0.411 | D=0.045 | <0.001 |
| anger | 0.049 | 0.216 | 0.075 | 0.263 | D=0.026 | <0.001 |
| fear | 0.426 | 0.495 | 0.404 | 0.491 | D=0.022 | <0.001 |
| sadness | 0.028 | 0.164 | 0.035 | 0.183 | D=0.007 | 0.408 |
| neutral | 0.236 | 0.425 | 0.271 | 0.444 | D=0.035 | <0.001 |
